# The white lupin trehalase gene *LaTRE1* regulates cluster root formation and function under phosphorus deficiency

**DOI:** 10.1093/plphys/kiae290

**Published:** 2024-05-28

**Authors:** Tianyu Xia, Xiaoqi Zhu, Yujie Zhan, Bowen Liu, Xiangxue Zhou, Qian Zhang, Weifeng Xu

**Affiliations:** Joint International Research Laboratory of Water and Nutrient in Crop and College of JunCao Science and Ecology, Fujian Agriculture and Forestry University, Jinshan Fuzhou 350002, China; Joint International Research Laboratory of Water and Nutrient in Crop and College of JunCao Science and Ecology, Fujian Agriculture and Forestry University, Jinshan Fuzhou 350002, China; Joint International Research Laboratory of Water and Nutrient in Crop and College of JunCao Science and Ecology, Fujian Agriculture and Forestry University, Jinshan Fuzhou 350002, China; Joint International Research Laboratory of Water and Nutrient in Crop and College of JunCao Science and Ecology, Fujian Agriculture and Forestry University, Jinshan Fuzhou 350002, China; Joint International Research Laboratory of Water and Nutrient in Crop and College of JunCao Science and Ecology, Fujian Agriculture and Forestry University, Jinshan Fuzhou 350002, China; Joint International Research Laboratory of Water and Nutrient in Crop and College of JunCao Science and Ecology, Fujian Agriculture and Forestry University, Jinshan Fuzhou 350002, China; Joint International Research Laboratory of Water and Nutrient in Crop and College of JunCao Science and Ecology, Fujian Agriculture and Forestry University, Jinshan Fuzhou 350002, China

## Abstract

Under phosphorus (P) deficiency, white lupin (*Lupinus albus* L.) forms a specialized root structure, called cluster root (CR), to improve soil exploration and nutrient acquisition. Sugar signaling is thought to play a vital role in the development of CR. Trehalose and its associated metabolites are the essential sugar signal molecules that link growth and development to carbon metabolism in plants; however, their roles in the control of CR are still unclear. Here, we investigated the function of the trehalose metabolism pathway by pharmacological and genetic manipulation of the activity of trehalase in white lupin, the only enzyme that degrades trehalose into glucose. Under P deficiency, validamycin A treatment, which inhibits trehalase, led to the accumulation of trehalose and promoted the formation of CR with enhanced organic acid production, whereas overexpression of the white lupin *TREHALASE1* (*LaTRE1*) led to decreased trehalose levels, lateral rootlet density, and organic acid production. Transcriptomic and virus-induced gene silencing results revealed that LaTRE1 negatively regulates the formation of CRs, at least partially, by the suppression of *LaLBD16*, whose putative ortholog in Arabidopsis (*Arabidopsis thaliana*) acts downstream of ARF7- and ARF19-dependent auxin signaling in lateral root formation. Overall, our findings provide an association between the trehalose metabolism gene *LaTRE1* and CR formation and function with respect to organic acid production in white lupin under P deficiency.

## Teaser

A trehalase enzyme that degrades trehalose into glucose modulates genes associated with lateral root initiation and carbon metabolism and thus regulates CR formation in white lupin.

## Introduction

Phosphorus (P) is one of the most limiting essential nutrients for plant growth and development. Plants take up P in the form of $H_{2}{\mathrm{PO}}_{4}^{-}$ and ${\mathrm{HPO}}_{4}^{2-}$, which in soils are often bound to metal cations such as Ca^2+^, Mg^2+^, and Al^3+^ or converted into organic forms not amenable for plant uptake (Schachtman et al. 1998). A range of strategies have evolved to increase P availability and uptake. The most common strategy employed by 82% of angiosperms is a symbiotic association with mycorrhizal fungi (Brundrett 2002). In addition, the formation of cluster root (CR) is thought to be another efficient strategy (Neumann and Martinoia 2002). CR is termed as a section of densely packed rootlets with determinate growth in a certain region along the lateral root (Meng et al. 2012). White lupin (*Lupinus albus* L.) is the most widely-used model plant for studies on CR morphology and function (Neumann and Martinoia 2002; Shane and Lambers 2005). Under P deficiency, white lupin produces CR to enhance spatial acquisition of the available fraction of sparingly soluble P by increasing the root absorption surface and releasing large amounts of organic acids (e.g. citrate and malate), flavonoids, protons, and acid phosphatases (Watt and Evans 1999b; Miller et al. 2001; Uhde-Stone et al. 2003; Shen et al. 2005). High-throughput approaches, such as transcriptomics, proteomics, and metabolomics, have identified many of the genes, proteins, and metabolites involved in CR formation and function and are beginning to reveal networks of gene expression, protein modification, and metabolic adaptation (Tian et al. 2009; Rodriguez-Medina et al. 2011; O’Rourke et al. 2013; Secco et al. 2014; Wang et al. 2014b; Müller et al. 2015; Hufnagel et al. 2020; Xu et al. 2020).

Sugars act as signal molecules that mediate plant growth, development, metabolism, and response to stimuli (Ruan 2014; Li and Sheen 2016). High light intensity and elevated atmospheric CO_2_ concentrations increased CR formation and carboxylate exudation under P deficiency, in agreement with sugar as a key stimulator of CR (Watt and Evans 1999a; Cheng et al. 2014). However, sucrose was able to establish CR morphology, but not function which is characterized by an intense temporal secretion of root exudates (e.g. citrate and malate), indicating that sugars other than sucrose could function as signaling molecules that regulate CR function (Wang et al. 2014a). A recent study showed that the level of trehalose 6-phosphate (Tre6P) is associated with light-dependent phosphorylation of phosphoenolpyruvate carboxylase (PEPC), and the latter encodes a key enzyme involved in CR function through controlling carbohydrate partitioning to organic acids (Shane et al. 2016). Tre6P, the intermediate of trehalose metabolism, is an essential signal metabolite that links the growth and development of plants to their metabolic status (Lunn et al. 2014). In plants, trehalose metabolism pathway contains 3 steps. First, Tre6P synthase (TPS) uses UDP-Glucose and Glucose 6-phosphate to produce Tre6P, which is followed by the conversion of trehalose via Tre6P phosphatase (TPP). Then, trehalose is hydrolyzed by trehalase (TRE) into 2 molecules of glucose (Paul et al. 2008). Genetic manipulation of microbial or plant trehalose metabolism genes caused alterations in growth, development, metabolism, and stress resistance in plants (Gómez et al. 2010; Van Houtte et al. 2013; Lunn et al. 2014; Figueroa et al. 2016; Ponnu et al. 2020). For example, overexpressing the *E. coli* TPS gene *otsA* in Arabidopsis (*Arabidopsis thaliana*) induced increases in Tre6P level, leading to phosphorylation of PEPC that promotes the carbon flux into organic acids (Figueroa et al. 2016); *AtTRE1*-overproducing plants had decreased trehalose level and recovered better after drought stress, whereas loss of *AtTRE1* function led to elevated trehalose level and a drought-susceptible phenotype (Van Houtte et al. 2013).

However, the role of trehalose metabolism genes in CR development is largely unknown. Herein, genes involved in trehalose metabolism in white lupin were identified, and by using pharmacological inhibition, *A. rhizogenes*-mediated gene overexpression and virus-induced gene silencing (VIGS) approaches, the function of *LaTRE1* was further investigated. Finally, our findings revealed that *LaTRE1* is involved in the trehalose metabolism pathway and is associated with the formation and function of CR in white lupin under P deficiency.

## Results

### LaTRE1 is the only enzyme for trehalose hydrolysis and is differentially expressed during the development of CR

**Figure 1.**
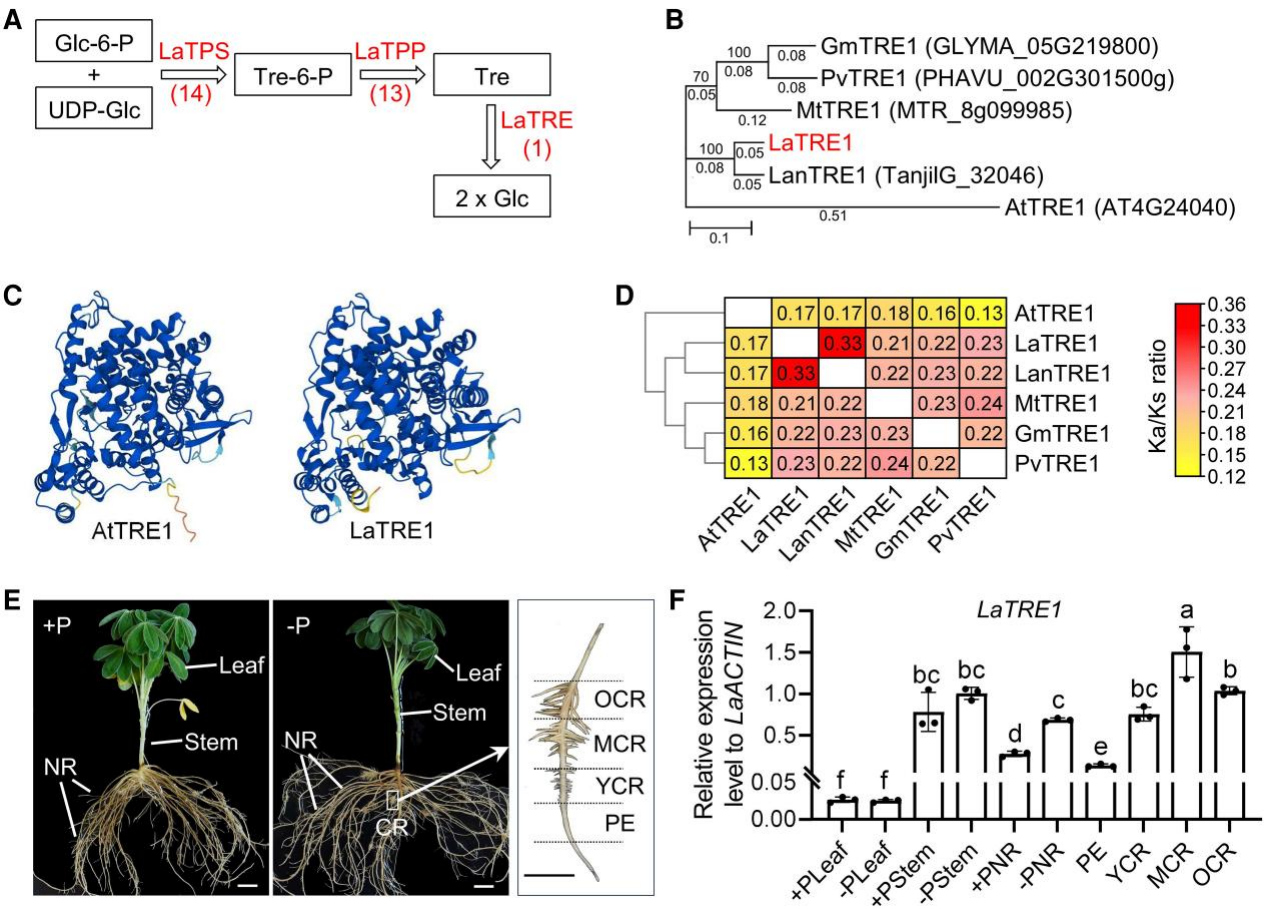
Molecular characterization of the trehalose metabolism gene *LaTRE1*. **A)** The pathway and enzymes of trehalose metabolism in plants. TPS, TREHALOSE-6-PHOSPHATE SYNTHASE; TPP, TREHALOSE-6-PHOSPHATE PHOSPHATASE; TRE, TREHALASE; Glc-6-P, glucose-6-phosphate; UDP-Glc, UDP-glucose; Tre6P, trehalose-6-phosphate; Tre, trehalose; and Glc, glucose. The number of *LaTPS*, *LaTPP,* and *LaTRE* in white lupin was indicated in parentheses. **B)** Phylogenetic analysis of TRE1 proteins in Arabidopsis (*A. thaliana*, At), common bean (*P. vulgaris*, Pv), barrel medic (*M. truncatula*, Mt), soybean (*G. max*, Gm), and narrow-leafed lupin (*L. angustifolius*, Lan). Tree scale bar represents distance as the number of substitutions per site. **C)** Alphafold-predicted structures of AtTRE1 and LaTRE1. **D)** The Ka/Ks ratios of *TRE1* gene pairs. **E)** Phenotypes of 4-wk-old white lupin plants grown under P-sufficient (+P) and -deficient (−P) conditions. Different types of root tissues were indicated, including NR under +P and −P conditions and 4 developmental stages of CR under −P condition (PE, YCR, MCR, and OCR). Bars = 2 cm for white lupin plants and 1 cm for CR. Images were digitally extracted. **F)** Relative expression levels of *LaTRE1* to the internal gene *LaACTIN* (*Lalb_Chr04g0257761*) in tissues indicated in **E)**. Error bars represent SE, *n* = 3 biological replicates. The significant differences were determined by a one-way ANOVA test (*P* < 0.05) and indicated by different lowercase letters.

To investigate the role of trehalose metabolism pathway in white lupin, we characterized the genes involved in trehalose biosynthesis and degradation in white lupin genome. Using AtTPS1 (AT1G78580), AtTPPA (AT5G51460), and AtTRE1 (AT4G24040) amino acid sequences as queries, a total of 14 *TPS* genes, 13 *TPP* genes, and 1 *TRE* gene were identified in white lupin genome, named as *LaTPS1* to *LaTPS14*, *LaTPP1* to *LaTPP13*, and *LaTRE1*, respectively, based on their order on the chromosomes (Fig. 1A; Supplementary Table S1). Phylogenetic analysis showed that *LaTPS* genes were divided into 2 classes, with Class I genes containing at least 18 exons and Class II genes containing 3 to 8 exons, and *LaTPP* genes were also divided into 2 classes with all genes containing 9 to 14 exons (Supplementary Fig. S1), which was consistent with the previous study (Lunn 2007). In contrast to the large families of *LaTPS* and *LaTPP* genes, only one *TRE* gene was identified in white lupin, and similar results were found in Arabidopsis, common bean (*Phaseolus vulgaris*), barrel medic (*Medicago truncatula*), soybean (*Glycine max*), and narrow-leafed lupin (*Lupinus angustifolius*) (Fig. 1B). Protein sequence alignment and structure prediction confirmed that TRE proteins were highly conserved among these 6 species (Fig. 1C; Supplementary Figs. S2 and S3). Next, pairwise nonsynonymous (Ka) and synonymous substitution rates (Ks) and the Ka/Ks ratio were calculated for the TRE protein-coding sequences in these 6 species. As shown in Fig. 1D, the Ka/Ks ratios ranged from 0.13 to 0.33, the highest ratio (0.33) was found between *LaTRE1* and *LanTRE1*, while the lowest ratio (0.13) occurred when comparing *AtTRE1* and *PvTRE1*, and all the Ka/Ks ratios were less than 1, suggesting that *TRE* genes were under purifying selection.

Further, tissue-specific expression patterns of *LaTRE1* were analyzed by reverse transcription quantitative PCR (RT-qPCR), including leaf, stem, and normal root (NR) of the 4-wk-old white lupin plants grown under P-sufficient or -deficient conditions and 4 developmental zones of CR under P-deficient condition (pre-emergent zone, PE; young cluster root, YCR; mature cluster root, MCR; and old cluster root, OCR) (Fig. 1E). As shown in Fig. 1F, *LaTRE1* was highly expressed in stem and root but was lowly expressed in leaf; P deficiency significantly stimulated the transcription of *LaTRE1* in roots (one-way ANOVA test, *P* < 0.05) but not in leaf and stem; during the development of CR, *LaTRE1* was lowest expressed in PE, followed by YCR, and was highest in MCR. These results suggested that *LaTRE1*, encoding a putative trehalase, was induced by P deficiency in the root and was differentially expressed during the development of CR.

### Inhibition of trehalase activity stimulates the formation of CR with increased organic acid production

Given that trehalase was encoded by a single-copy of gene in white lupin genome, we manipulated the trehalase activity using pharmacological inhibition to preliminary investigate the role of trehalose metabolism pathway in white lupin. Validamycin A (β-D-glucopyranosilvalidoxylamine, ValA) is a widely used inhibitor of trehalase in previous studies (Goddijn et al. 1997; Müller et al. 2001; Sun et al. 2022). We first investigated whether ValA effectively inhibits endogenous trehalase activity in white lupin plants. One-week P-sufficient pre-cultured white lupin plants were transferred in P-sufficient or -deficient cultures with or without 200 *μ*M ValA for 1 wk, samples of total roots were then collected and analyzed for trehalase activity. Compared with those in the ValA-untreated roots, about 36% of trehalase activity was reduced in ValA-treated roots under P-sufficient condition, and 70% of trehalase activity was reduced under P deficiency (Supplementary Fig. S4), suggesting that ValA effectively inhibits the activity of trehalase in white lupin roots. In addition, trehalase activity in ValA-untreated roots was induced by P starvation (Supplementary Fig. S4), which was consistent with the expression data in Fig. 1F.

**Figure 2.**
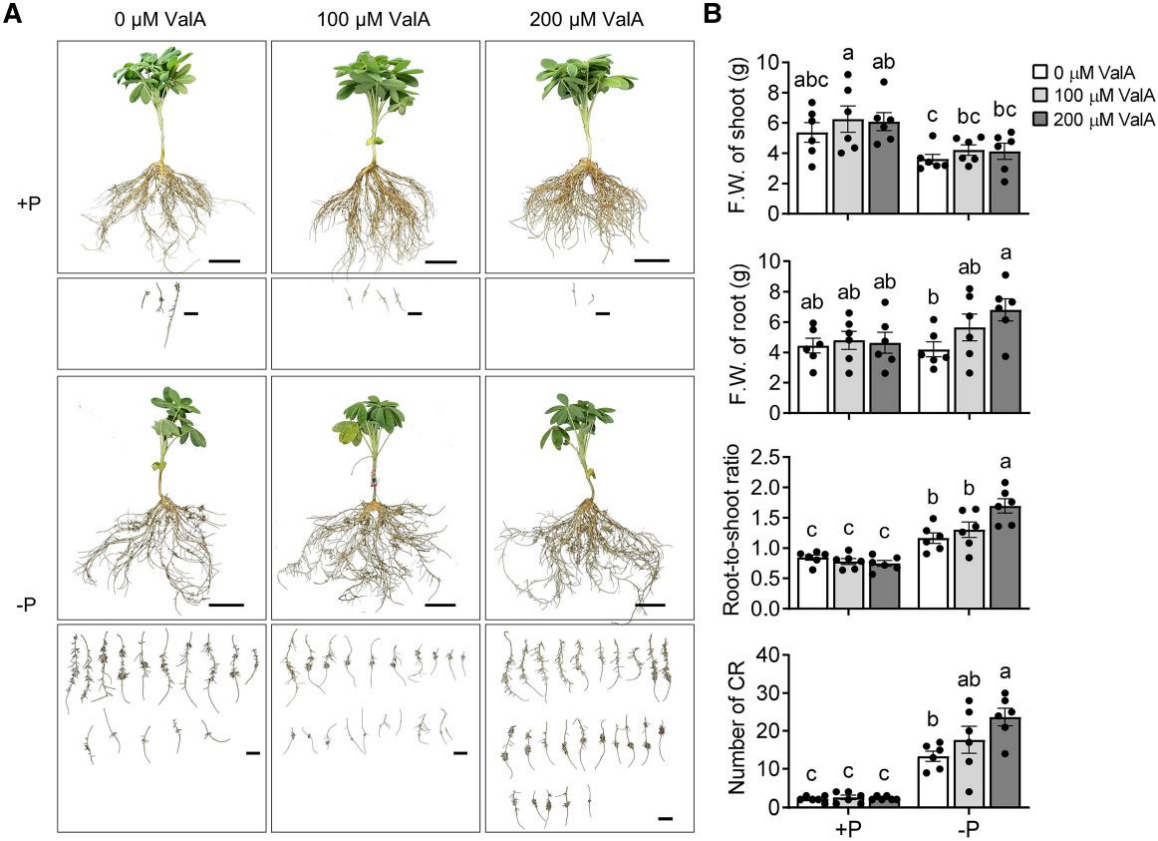
ValA treatment promotes the formation of CR under P deficiency. **A)** Phenotypes of 1-wk-old white lupin plants transplanted to +P and −P hydroponic cultures with 0, 100, and 200 *μ*M ValA for 3 wk, bars = 5 cm. The corresponding CRs of each plant were shown below, bars = 2 cm. Images were digitally extracted for comparison. **B)** F.W. of shoot and root, ratio of root-to-shoot fresh biomass, and number of CR per 4-wk-old white lupin plant grown under +P and −P solutions with 0, 100, and 200 *μ*M ValA. Error bars represent SE, *n* = 6 individual plants. The significant differences were determined by a one-way ANOVA test (*P* < 0.05) and indicated by different lowercase letters.

We next investigated the phenotypic changes occurring in white lupin plants treated with ValA. One-week-old plants grown in P-sufficient condition were transferred into P-sufficient or -deficient hydroponic solutions with different concentrations of ValA. After 3 wk of cultivation, only a small number of CRs appeared under P-sufficient control, and ValA treatment did not affect the fresh biomass of shoot and root nor the formation of CR, whereas the number of CR, fresh weight (F.W.) of root, and the ratio of root-to-shoot fresh biomass were significantly increased in P-deficient condition than those under P-sufficient condition, and these inductions were enhanced progressively with the increase in ValA concentrations (Fig. 2), suggesting that ValA treatment to inhibit trehalase activity promotes the formation of CR under P deficiency.

**Figure 3.**
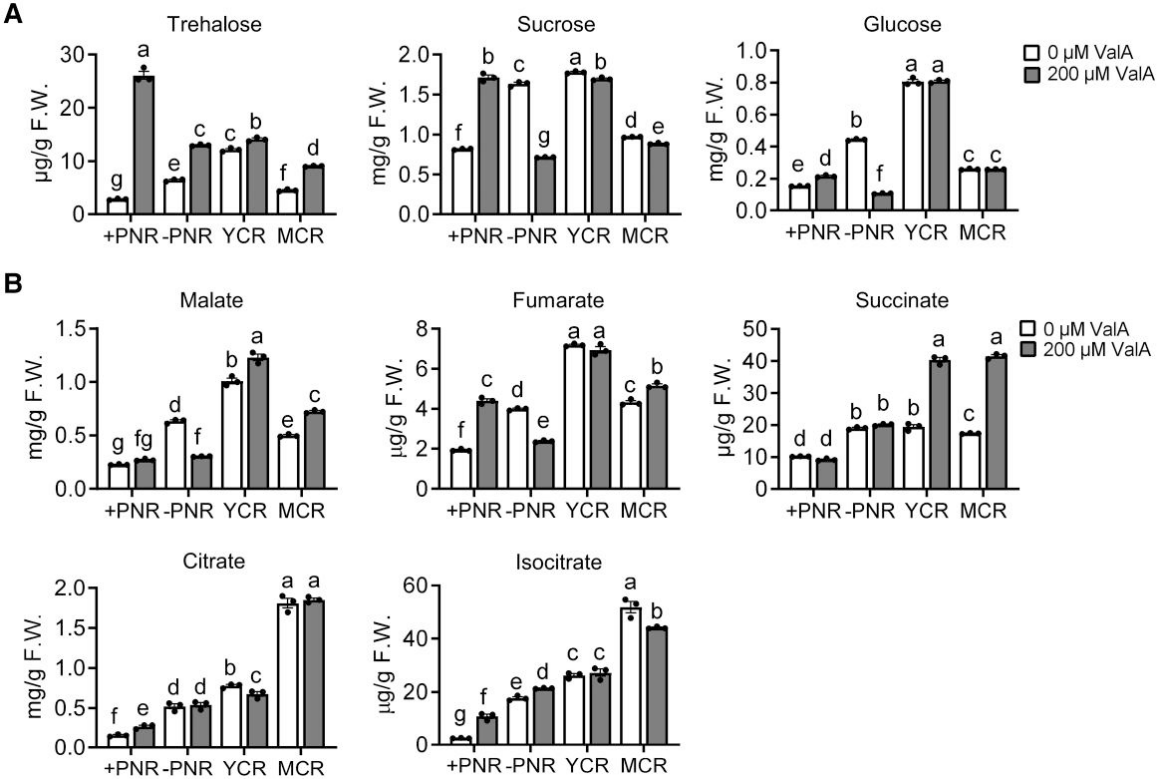
ValA treatment promotes the accumulation of trehalose and organic acids in CR. **A)** Contents of trehalose, sucrose, and glucose in different types of roots with 0 and 200 *μ*M ValA treatment. **B)** Contents of malate, fumarate, succinate, citrate, and isocitrate in different types of roots with 0 and 200 *μ*M ValA treatment. Error bars represent SE, *n* = 3 biological replicates. The significant differences were determined by a one-way ANOVA test (*P* < 0.05) and indicated by different lowercase letters. Source data of **A)** and **B)** were provided in **Supplementary Table S2**.

P deficiency-induced organic acid (e.g. malate and citrate) production and extrusion that help to solubilize P in soil are the typical physiological responses in YCR and MCR in white lupin (Neumann et al. 1999). Trehalose metabolism pathway has an especially large impact on the flux of carbon to organic acids (Figueroa et al. 2016). To investigate the effect of ValA treatment on the carbon metabolism of white lupin, especially in CR, the levels of sugar metabolites and organic acids were measured in ValA-treated and -untreated root tissues, including +PNR, −PNR, YCR, and MCR in Fig. 1E. Without ValA treatment, the amounts of trehalose, sucrose, and glucose were induced under P deficiency, and highest in YCR, followed by −PNR and MCR; with 200 *μ*M ValA treatment, an increase in trehalose level was noted in all 4 types of root tissues, the sucrose level was increased in +PNR and decreased in −PNR, YCR, and MCR, and the glucose level was increased in +PNR and decreased in −PNR but unchanged in YCR and MCR (Fig. 3A; Supplementary Table S2). For organic acids, without ValA treatment, the levels of malate, fumarate, succinate, citrate, and isocitrate were induced by P deficiency, in consistent with previous report (Müller et al. 2015); with ValA treatment, these inductions, except for citrate and isocitrate, were enhanced in YCR and/or MCR (Fig. 3B; Supplementary Table S2). Overall, these findings suggested that ValA treatment to inhibit trehalase activity leads to increased trehalose and promotes organic acid production in CR under P deficiency.

### Overexpression of *LaTRE1* leads to decreased lateral rootlet density and altered organic and amino acid metabolism

**Figure 4.**
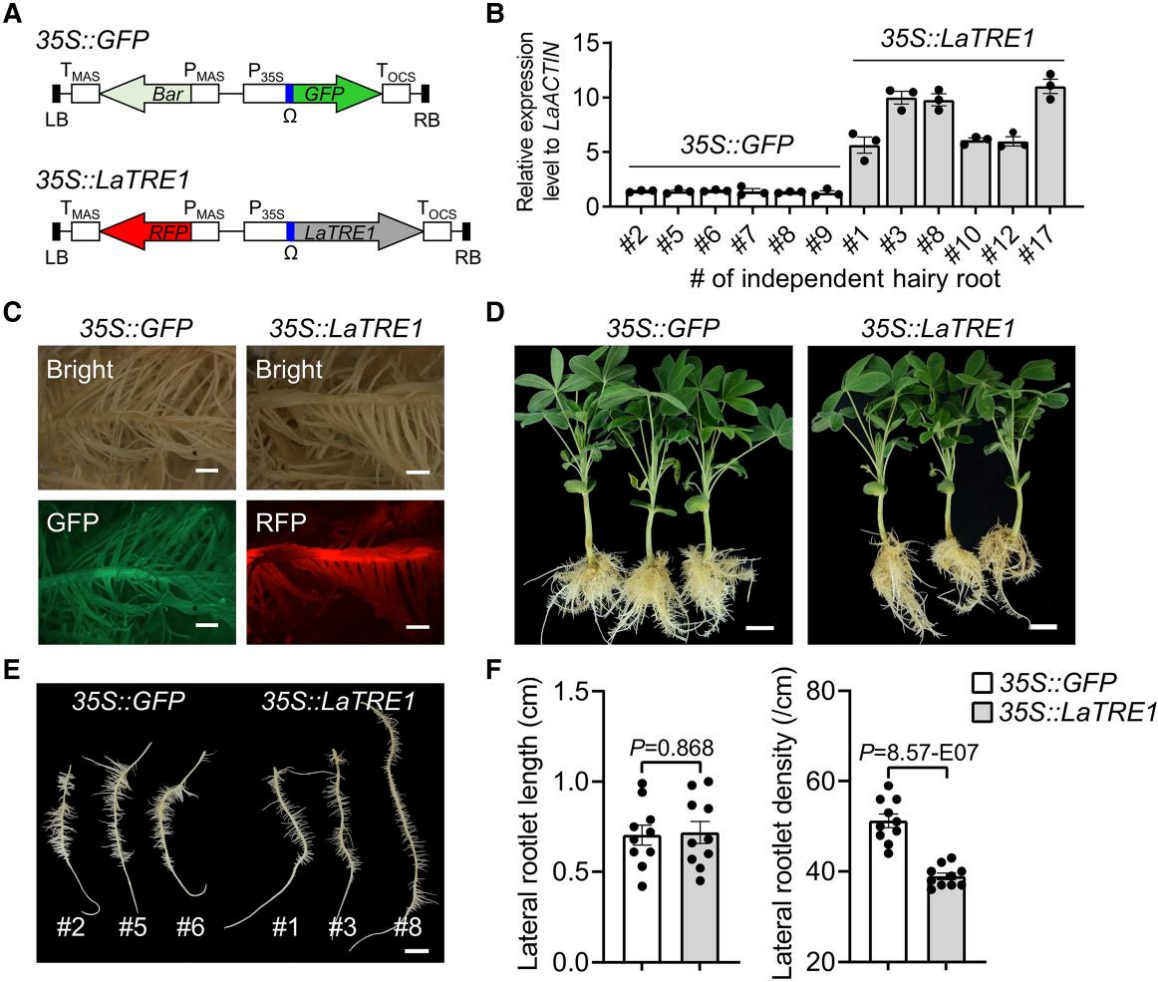
Overexpression of *LaTRE1* reduces the lateral rootlet density of hairy roots. **A)** Schematic presentation of *35S::GFP* and *35S::LaTRE1* constructs. **B)** The relative expression levels of *LaTRE1* gene to the internal control *LaACTIN* in *35S::GFP* and *35S::LaTRE1* transgenic roots. Each individual transgenic root was considered as an independent line and indicated as #2, #5, and so on. **C)** Positive transgenic hairy roots with green or red fluorescent signals, bars = 2 mm. **D)** Gross morphologies of the composite plants with *35S::GFP* and *35S::LaTRE1* transgenic roots grown under P deficiency, bars = 2 cm. Images were digitally extracted for comparison. **E)** Phenotypes of *35S::GFP* and *35S::LaTRE1* independent transgenic roots, bar = 1 cm. Images were digitally extracted for comparison. **F)** The average length and density of lateral rootlets per independent transgenic root. Error bars represent SE, *n* = 3 technical replicates for **B)** and 10 independent transgenic roots for **F)**, the significant differences were determined by unpaired 2-sided Student's *t*-test (*P* < 0.05).

In order to better understand the function of *LaTRE1* in white lupin, the composite plants with transgenic roots expressing *LaTRE1* under the control of *35S* promoter were generated, and the composite plants transformed with *35S::GFP* construct were used as controls (Fig. 4A). Positive transgenic roots were selected for fluorescent signals and validated by RT-qPCR for the overexpression of *LaTRE1* (Fig. 4, B and C). Since the effects of trehalase inhibitor on CR depend on the absence of P, the composite plants grown under P deficiency for 3 wk were used for further analyses. Within the whole culture period, there was no visual difference in the shoot between *GFP-* and *LaTRE1-*overexpressing composite plants (Fig. 4D). Detailed analysis of the transgenic root morphology of *35S::LaTRE1* and *35S::GFP* composite plants was conducted. However, *A. rhizogenes*-based transformation led to the development of “hairy-root” phenotype characterized by a high degree of lateral branching (Chilton et al. 1982; Collier et al. 2005), which makes us difficult to visually determine the typical CR phenotypes as shown in Fig. 1E. Therefore, only the length and density of lateral rootlet of transgenic roots were analyzed. We found that overexpression of *LaTRE1* led to a comparable lateral rootlet length and lower lateral rootlet density than the controls (Fig. 4, E and F), implicating a role of *LaTRE1* in the regulation of root morphology in white lupin under P deficiency.

**Figure 5.**
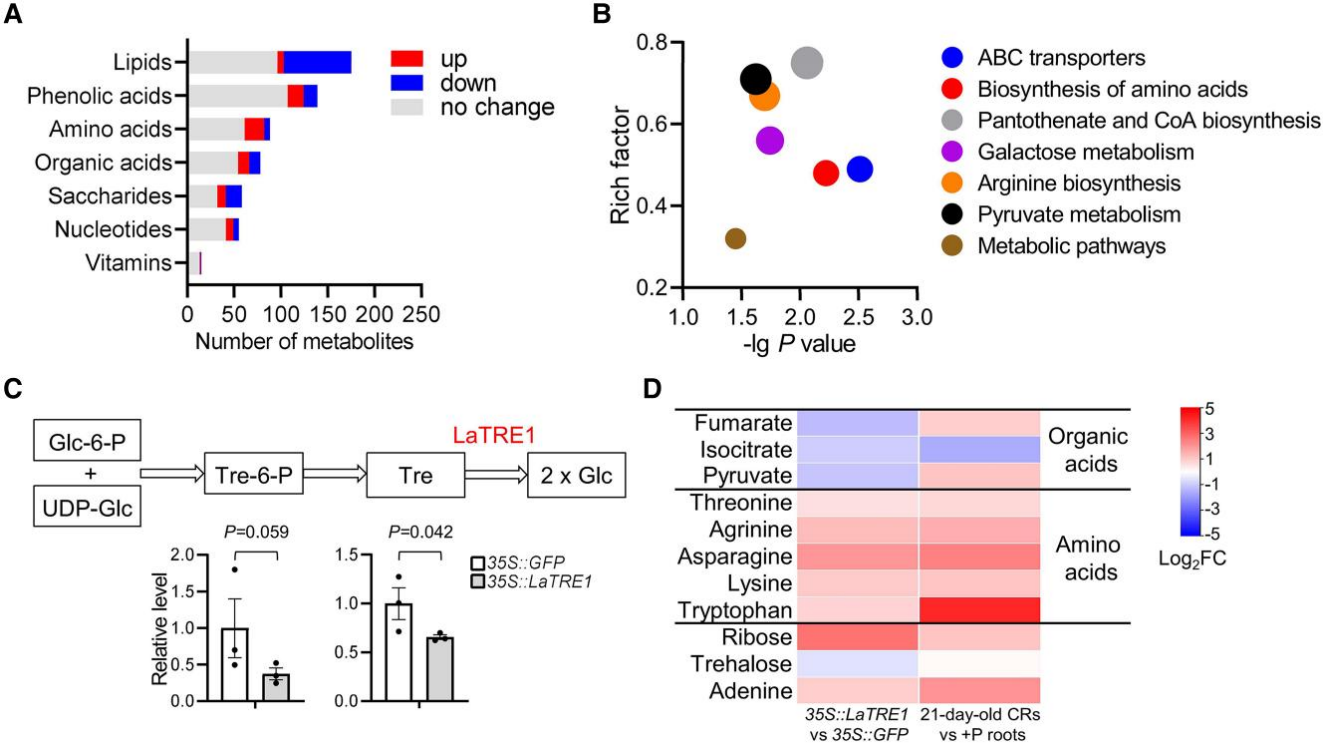
Metabolomic profiling of *35S::GFP* and *35S::LaTRE1* transgenic roots under P deficiency. **A)** Number of unchanged (gray), upregulated (red), and downregulated (blue) metabolites in *35S::LaTRE1* transgenic roots compared to the controls. **B)** KEGG enrichment analysis of the DAMs in *35S::LaTRE1* transgenic roots compared to the controls. **C)** Relative contents of Tre6P and trehalose in *35S::LaTRE1* transgenic roots compared to the controls. Error bars represent SE, *n* = 3 biological replicates, the significant differences were determined by unpaired 2-sided Student's *t* test (*P* < 0.05). UDP-Glc, UDP-glucose; Tre6P, trehalose-6-phosphate; Tre, trehalose; and Glc, glucose. **D)** Metabolites both changed in *35S::LaTRE1* transgenic roots compared to *35S::GFP* and in 21-d-old CRs compared to +P roots (Müller et al. 2015). FC, fold change. Source data of **A)**, **B)**, and **D)** were provided in Supplementary Tables S3 and S4.

Next, untargeted metabolome analysis was conducted to systematically investigate the metabolic changes between *35S::LaTRE1* and *35S::GFP* transgenic roots under P deficiency (See Materials and Methods for detail). In total, 608 metabolites covered 7 categories were detected, including lipids, phenolic acids, amino acids, organic acids, saccharides, nucleotides, and vitamins, and under the significance thresholds of fold change (FC) ≥ 1.3 or ≤ 1/1.3, variable importance in projection (VIP) ≥ 1 and *P* < 0.05, a total of 204 differentially accumulated metabolites (DAMs) were identified between *35S::LaTRE1* and *35S::GFP* (Supplementary Table S3). Among these DAMs, the amounts of 91% lipids (72 of 79) and 60% saccharides (17 of 26) were decreased, whereas the amounts of 78% amino acids (21 of 27) were increased (Fig. 5A). Kyoto Encyclopedia of Genes and Genomes (KEGG) pathway enrichment analysis showed that these DAMs were mainly enriched in “ABC transporters”, “Biosynthesis of amino acids”, “Galactose metabolism”, and “Pyruvate metabolism” (Fig. 5B; Supplementary Table S4). Moreover, in consistent with the putative trehalose hydrolyzing activity of LaTRE1, the trehalose level was significantly decreased in *LaTRE1*-overproducing roots compared with that in the controls (0.66-FC; Student's *t*-test, *P* < 0.05); and about 0.38-fold decrease in the amounts of Tre6P was also observed, although the *P-*value was slightly greater than the threshold (Student's *t*-test, *P* = 0.059 > 0.05) (Fig. 5C). Compared with the public P-starvation response (PSR) metabolic profiles in CR (Müller et al. 2015), we found that 24 PSR-metabolites in CR were detected in our metabolic data, and 11 of them were differentially accumulated after *LaTRE1*-overexpression (Supplementary Table S3). As shown in Fig. 5D, all 5 P-starvation-induced amino acids were induced by *LaTRE1*-overexpression, whereas 2 of 3 P-starvation-induced organic acids (fumarate and pyruvate) were decreased after *LaTRE1*-overexpression. These results suggested that *LaTRE1* is involved in the trehalose metabolism pathway and overexpression of *LaTRE1* leads to the alteration of root morphology and organic and amino acid metabolism.

### Genes involved in lateral root formation and organic and amino acid metabolism were regulated by *LaTRE1*-overexpression

**Figure 6.**
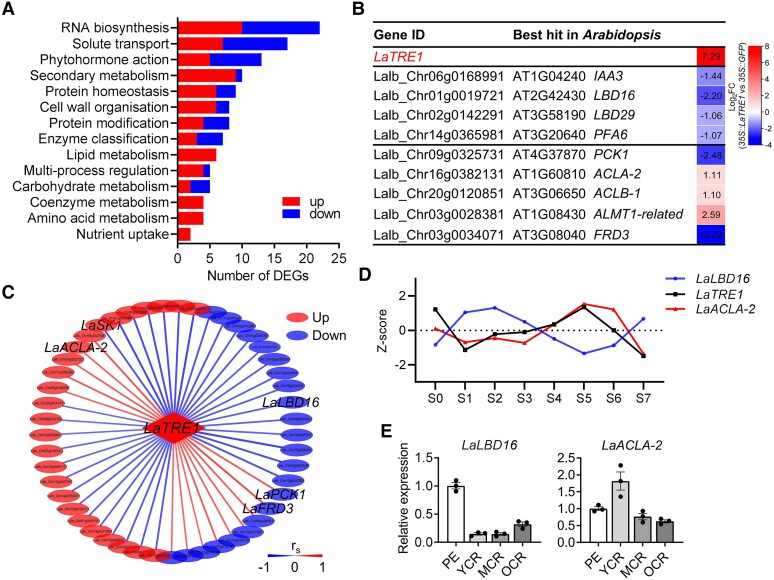
Transcriptomic profiling of *35S::GFP* and *35S::LaTRE1* transgenic roots under P deficiency. **A)** Mapman functional classification of the DEGs in *LaTRE1*-overexpression roots compared to the controls. **B)** The DEGs involved in lateral root formation, organic acid production, and excretion. **C)** Co-expression network between *LaTRE1* and the DEGs during the development of CR. Spearman’s correlation coefficients (*r_s_*) between *LaTRE1* and the DEGs during CR development were calculated according to the public transcriptomic datasets that cover 8 developmental zones of CR (Hufnagel et al. 2020). **D)** Relative expression levels of *LaTRE1*, *LaLBD16*, and *LaACLA-2* in the 8 developmental zones of CRs (Supplementary S0 to S7). **E)** RT-qPCR validation of *LaLBD16* and *LaACLA-2* expression patterns at different stages of CR, including PE, YCR, MCR, and OCR. Source data of **A)**, **B)**, and **C)** were provided in Supplementary Tables S6 to S8.

To uncover the molecular basis of the phenotypic and metabolic changes caused by *LaTRE1*-overexpression, transgenic roots of *LaTRE1*-overproducing composite plants grown under P deficiency for 3 wk were collected for transcriptome analysis, whereas the *35S::GFP* transgenic roots were used as controls. Under the significance thresholds of |log_2_FC| ≥ 1, *P* < 0.05 and false discovery rate (FDR) < 0.2, a total of 231 differentially expressed genes (DEGs) were identified, including 140 upregulated and 91 downregulated genes (Supplementary Table S5). Mapman functional annotation and classification revealed that these DEGs are mainly assigned to RNA biosynthesis, phytohormone action, solute transport, carbohydrate metabolism, and amino acid metabolism processes (Fig. 6A; Supplementary Table S6). Moreover, there was a 156-fold increase of *LaTRE1* transcripts in *35S::LaTRE1* transgenic roots than the controls, and genes associated with lateral root formation and organic acid metabolism and excretion were differentially expressed after *LaTRE1*-overexpression (Fig. 6B; Supplementary Table S7), including the putative lupin orthologs of *AUXIN/INDOLE-3-ACETIC ACID3* (*IAA3*) (Goh et al. 2012), *LATERAL ORGAN BOUNDARIES-DOMAIN16* (*LBD16*) and *LBD29* (Okushima et al. 2007), *PERICYCLE FACTOR TYPE-A*6 (*PFA6*) (Zhang et al. 2021), *PHOSPHOENOLPYRUVATE CARBOXYKINASE1* (*PCK1*) (Penfield et al. 2012), *Al-ACTIVATED MALATE TRANSPORTER1* (*ALMT1*) (Hoekenga et al. 2006), and *FERRIC REDUCTASE DEFECTIVE3* (*FRD3*) (Durrett et al. 2007) in Arabidopsis and *ATP-DEPENDENT CITRATE LYASE A-2* (*ACLA-2*) and *ACLB-1* in white lupin (Langlade et al. 2002).

Further, based on the transcriptomic datasets that cover 8 developmental zones of CR (Hufnagel et al. 2020), we found that expression patterns of 49 DEGs in our transcriptomic data showed a high correlation with *LaTRE1* during the development of CR (Spearman’s correlation analysis, |*r_s_*| > 0.7, *P* < 0.05), including the putative lupin orthologs of *LBD16*, *ACLA-2*, *PCK1*, and *FRD3* mentioned above (Fig. 6C; Supplementary Table S8). Previous studies showed that *LBD16* gene was involved in the auxin-mediated lateral root formation in Arabidopsis (Okushima et al. 2007), and the activity ATP-citrate lyase (ACL) encoded by the *ACLA* and *ACLB* genes of white lupin was implicated in malate metabolism and excretion in juvenile CR (Langlade et al. 2002). We found that *LaLBD16* was downregulated by *LaTRE1*-overexpression and showed a strong negative correlation with *LaTRE1* during the development of CR (*r_s_* = −0.79, *P* = 0.02), whereas *LaACLA-2* was upregulated by *LaTRE1*-overexpression and showed a strong positive correlation with *LaTRE1* during the development of CR (*r_s_* = 0.76, *P* = 0.03) (Fig. 6, C and D). Further, expression levels of *LaLBD16* and *LaACLA-2* were validated by RT-qPCR analyses during the development of CR (Fig. 6E) and in *35S::LaTRE1* and *35S::GFP* transgenic roots (Supplementary Fig. S5A and B).

**Figure 7.**
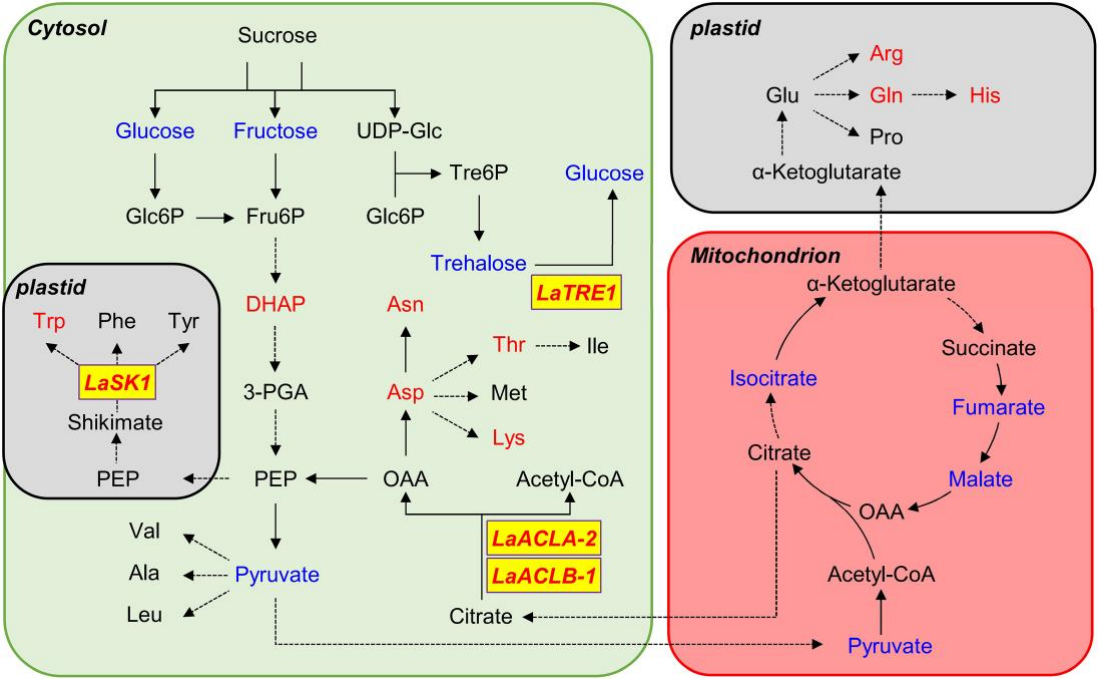
Overexpression of *LaTRE1* leads to the induction of amino acids and reduction of sugars and organic acids in central metabolism (See also Supplementary fig. S5). Metabolites together with key genes upregulated and downregulated by *LaTRE1*-overexpression were marked in red and blue, respectively. Solid arrows represent single-enzyme reactions and dashed arrows represent multiple reactions or transport between compartments. Source data were provided in Supplementary Tables S3 and S5.

To better understand the molecular basis of metabolic changes regulated by *LaTRE1*, we showed genes and metabolites upregulated and downregulated by *LaTRE1*-overexpression on a map of central metabolism (Fig. 7). In agreement with its function and previous study in Arabidopsis (Van Houtte et al. 2013), overexpression of *LaTRE1* led to decreased trehalose levels (Fig. 7). The amounts of the glycolysis intermediates such as glucose, fructose, and pyruvate and the tricarboxylic acid cycle intermediates such as malate, fumarate, and isocitrate were downregulated by *LaTRE1*-overexpression, whereas the amounts of amino acids such as arginine, glutamine, histidine, tryptophan, asparagine, aspartate, threonine, and lysine were upregulated by *LaTRE1*-overexpression (Fig. 7; Fig. 5C). Previous studies have shown that the cytosol-localized ACL, encoded by *ACLA* and *ACLB* genes of Arabidopsis, catalyzes the conversion of citrate to acetyl-CoA and oxaloacetic acid (OAA), which the latter may subsequently be transaminated to aspartate (Fatland et al. 2005; Eastmond et al. 2015), and *SK1* encodes a plastid-localized shikimate kinase that participates in the shikimate pathway, a common route leading to the production of tryptophan, tyrosine, and phenylalanine (Herrmann and Weaver 1999). We found that the putative lupin orthologs of *ACLA-2*, *ACLB-1*, and *SK1* were induced by *LaTRE1*-overproducing (Fig. 7; Supplementary Fig. S5A and B), suggesting that the accumulation of amino acids deriving from OAA and shikimate after *LaTRE1*-overexpression might be associated with the upregulation of *LaACLA-2*, *LaACLB-1*, and *LaSK1*.

### *LaTRE1* negatively regulates the formation of CR by repressing *LaLBD16*

**Figure 8.**
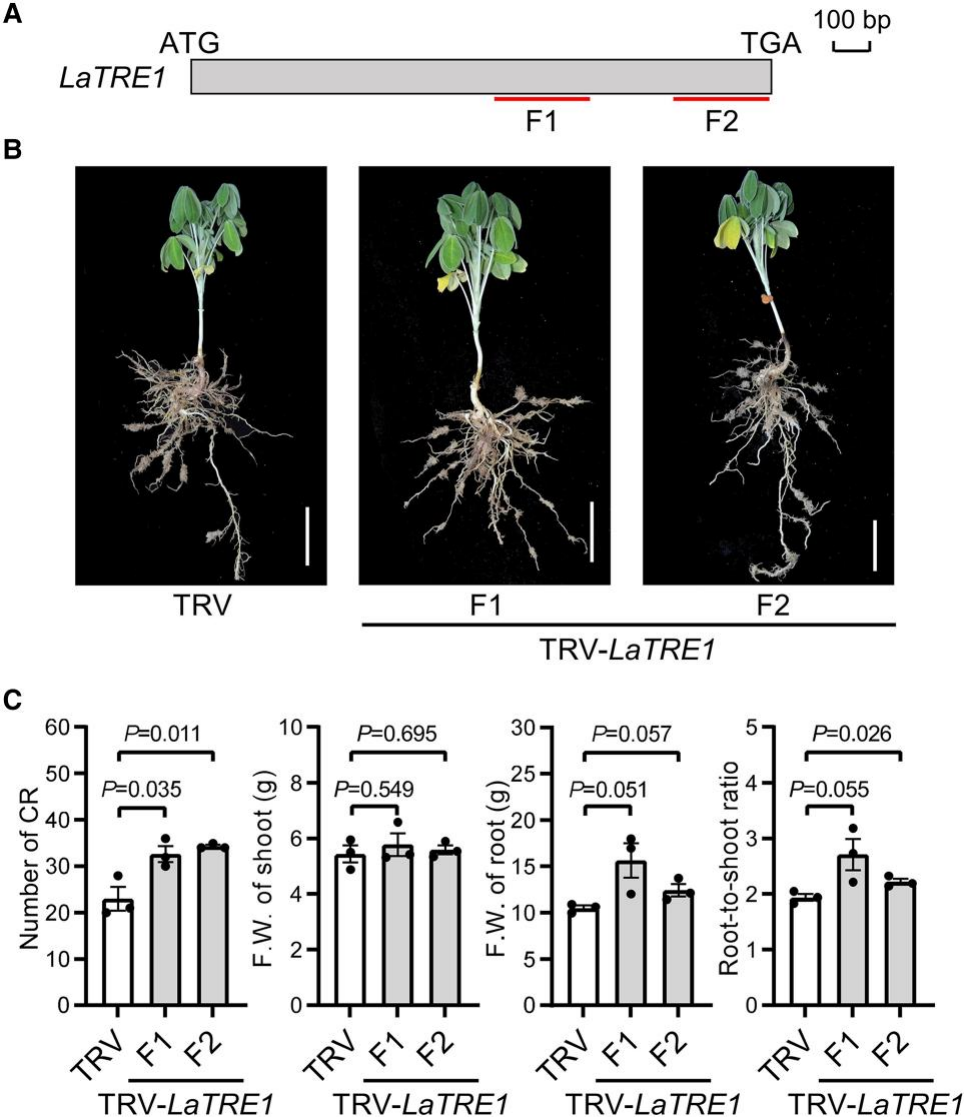
Silencing of *LaTRE1* promotes the formation of CR under p deficiency. **A)** Schematic representation of the gene-specific fragments (F1 and F2) of *LaTRE1* for construction of the TRV-*LaTRE1* vectors. **B)** Gross morphologies of empty vector control (TRV) and 2 independent *LaTRE1*-silenced lines generated by targeting F1 and F2 in **A)**, bars = 5 cm. Images were digitally extracted for comparison. **C)** Number of CR, F.W. of shoot and root, ratio of root-to-shoot fresh biomass of empty vector controls (TRV) and 2 independent *LaTRE1*-silenced lines. Error bars represent SE, *n* = 3 individual plants, the significant differences were determined by unpaired 2-sided Student's *t* test (*P* < 0.05).

Tobacco rattle virus-induced gene silencing (TRV-VIGS) assay was applied to further investigate the function of *LaTRE1* in CR. Two ∼300-bp fragments (F1 and F2) of protein-coding sequence of *LaTRE1* were used to knock down its expression in white lupin roots independently (Fig. 8A). RT-qPCR results showed that *LaTRE1* expression was successfully reduced in both independent *LaTRE1*-silenced lines (Supplementary Fig. S6). Under P deficiency, the *LaTRE1*-silenced plants of both independent lines exhibited a significant increase in CR number, a comparable shoot F.W., and a slight increase in root F.W. compared with the empty vector controls (TRV) (Student's *t*-test, *P* < 0.05), leading to increased root-to-shoot fresh biomass ratio (Fig. 8, B and C). In addition, expression levels of the *LaLBD16* gene were upregulated in both independent *LaTRE1*-suppression lines (Supplementary Fig. S6). These results suggest a negative effect of *LaTRE1* on the formation of CR and expression of *LaLBD16*.

**Figure 9.**
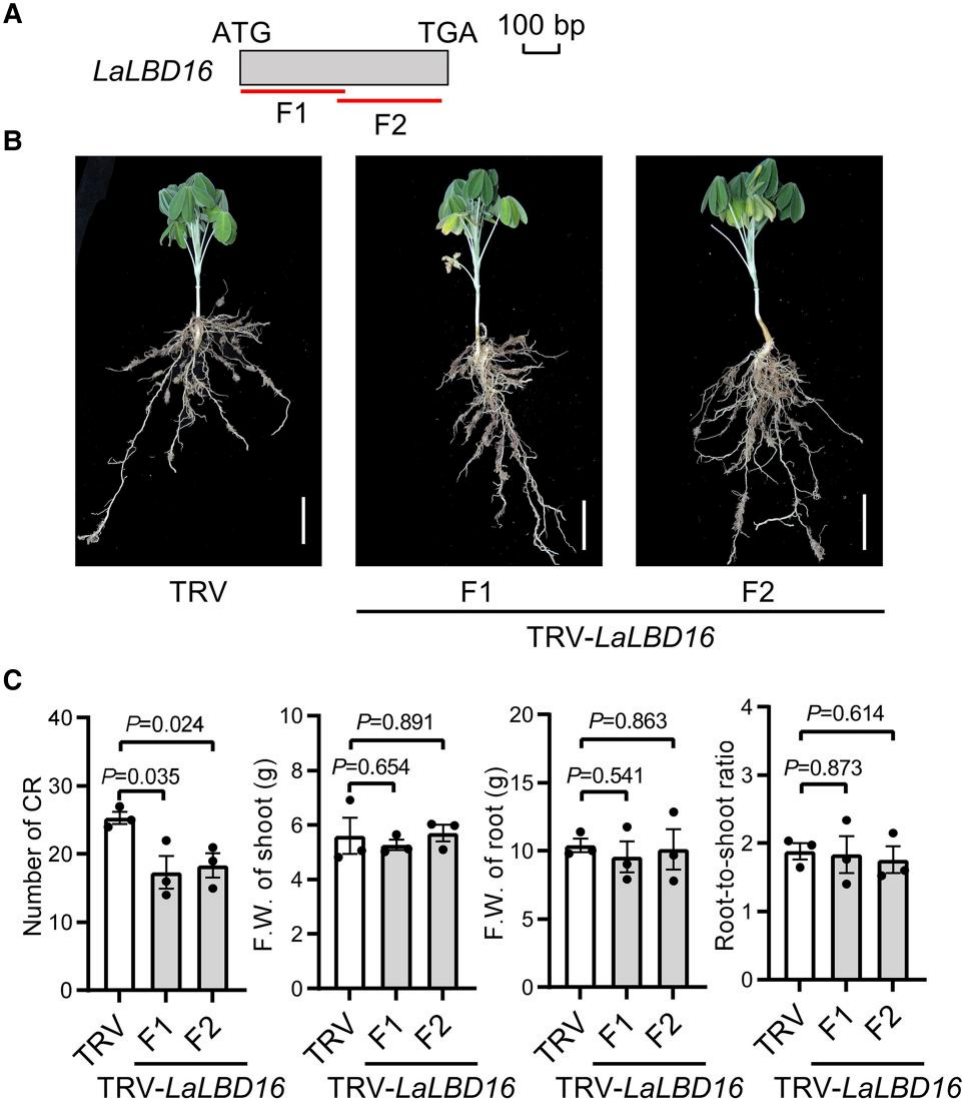
Silencing of *LaLBD16* impacts the formation of CR under P deficiency. **A)** Schematic representation of the gene-specific fragments (F1 and F2) of *LaLBD16* for construction of the TRV-*LaLBD16* vectors. **B)** Gross morphologies of empty vector control (TRV) and 2 independent *LaLBD16*-silenced lines generated by targeting F1 and F2 in **A)**, bars = 5 cm. Images were digitally extracted for comparison. **C)** Number of CR, F.W. of shoot and root, ratio of root-to-shoot fresh biomass of empty vector controls (TRV), and 2 independent *LaLBD16*-silenced lines. Error bars represent SE, *n* = 3 individual plants, the significant differences were determined by unpaired 2-sided Student's *t*-test (*P* < 0.05).

Given the important role of the putative Arabidopsis ortholog of *LaLBD16* in lateral root formation (Okushima et al. 2007), we thus investigated whether the increased CR number in *LaTRE1*-silenced plants could be associated with the downregulation of *LaLBD16*. Two independent TRV-VIGS experiments that target different regions of *LaLBD16* protein coding sequence were performed (Fig. 9A). RT-qPCR showed that the *LaLBD16* transcript abundances were successfully reduced in both independent *LaLBD16*-silenced lines (Supplementary Fig. S7). Phenotypic analysis showed that the *LaLBD16*-silenced plants of both independent lines displayed a significant decrease in CR number and a comparable shoot or root F.W. than the controls (Student's *t*-test, *P* < 0.05) (Fig. 9, B and C), and expression levels of *LaTRE1* were unchanged in the *LaLBD16*-silenced roots compared with the controls (Supplementary Fig. S7). These results supported the idea that *LaTRE1* negatively regulates the formation of CR, at least partially, by suppressing the expression of *LaLBD16*.

## Discussion

### *LaTRE1* involves in the trehalose metabolism pathway

There are 5 known trehalose metabolism pathways, but the most common pathway, and the only one present in plants is a 3-step pathway catalyzed by TPS, TPP, and TRE (Paul et al. 2008). In contrast to the large *TPS* and *TPP* gene families, the *TRE* gene contains only 1 copy in Arabidopsis, white lupin, and other 4 examined legumes (Fig. 1B). In plants, trehalases were implicated in the regulation of growth, carbon metabolism, and abiotic stress response. In Arabidopsis, overexpression of *AtTRE1*, which can complement the loss of function of the acid trehalase in yeast (Müller et al. 2001; Frison et al. 2007), led to increased trehalose contents and a drought-resistant phenotype, whereas mutation of *AtTRE1* led to increased trehalose contents and a drought-susceptible phenotype (Van Houtte et al. 2013); in barrel medic, *MtTRE1* was highly expressed in root nodules and downregulated under salt stress (López et al. 2008), and ValA-induced trehalose accumulation in root nodules improved the response of plants to salt stress (López et al. 2009); and in common bean, downregulation of *PvTRE1* led to increased trehalose contents, bacteroid number, nitrogenase activity, and nodule biomass, and transcript accumulation of genes involved in growth, carbon metabolism, nitrogen assimilation, and autophagy was enhanced too (Barraza et al. 2013). Protein alignment and structure prediction showed a high degree of sequence conservation and perhaps of structure/function identity between LaTRE1 and other plant trehalases (Supplementary Figs. S2 and S3). Herein, *LaTRE1* was a response to P deficiency in roots and differentially expressed during the development of CR (Fig. 1F); ValA treatment to inhibit trehalase activity led to increased trehalose content, CR number, and organic acid production (Figs. 2 and 3); overexpression of *LaTRE1* led to decreased trehalose levels, lateral rootlet density, and organic acid production (Figs. 4 and 5), whereas silencing of *LaTRE1* led to increased CR number (Fig. 8). All these results suggested that *LaTRE1* is involved in the trehalose metabolism pathway and plays an important role in the formation and carbon metabolism of white lupin CR under P deficiency.

### *LaTRE1* negatively regulates the formation of CR

Several attempts to investigate the role of trehalose metabolism pathway in plants were performed by overexpressing bacterial, fungal, or endogenous genes involved in trehalose metabolism or pharmacologically inhibiting endogenous trehalase activity, suggesting the function of trehalose metabolism pathway in root nodule development, salt stress response, carbon metabolism, drought tolerance, and phytohormone action (e.g. abscisic acid and auxin) (Goddijn et al. 1997; Romero et al. 1997; Avonce et al. 2004; López et al. 2009; Van Houtte et al. 2013; Figueroa et al. 2016; Wang et al. 2020; Morales-Herrera et al. 2023). Recent reports have shown that Arabidopsis *TPPB* is differentially expressed during lateral root formation, and *tppb* mutants had increased lateral root branching density, while *TPPB* overexpression lines exhibited an opposite phenotype (Morales-Herrera et al. 2023), suggesting that the trehalose metabolism pathway plays an important role in lateral root development. Herein, under P deficiency, ValA treatment to inhibit trehalase activity and silencing of *LaTRE1* promoted the formation of CR (Figs. 2 and 8), while *A. rhizogenes*-mediated *LaTRE1* overexpression led to decreased lateral rootlet density of transgenic roots (Fig. 4), suggesting that *LaTRE1* negatively regulates the formation of CR in response to P starvation.

A number of studies have shown that phytohormones play important roles in root growth and development in response to P starvation (Sun et al. 2016). For example, *GmRR1* encodes a soybean B-type response regulator and involves in plants response to low-P stress by modifying root architecture through crosstalk among auxin, ethylene, and cytokinin signaling pathways (Yang et al. 2024). Consistent with this finding, several clues supported the view that *LaTRE1* might regulate the formation of CR under P deprivation via a mechanism involving auxin. First, genes associated with auxin action were differentially expressed in *LaTRE1*-overexpression roots compared with the controls, and a slight but not significant increase in IAA level was observed (Student's *t*-test, *P* = 0.15 > 0.05) (Supplementary Fig. S8). Second, transcriptomic and VIGS-silencing experiments revealed that *LaTRE1* exerts the negative effect on CR formation probably by repressing *LaLBD16* (Figs. 6, 8 and 9), whose putative ortholog acts downstream of AUXIN RESPONSE FACTOR7 (ARF7) and ARF19-dependent auxin signaling in lateral root formation in Arabidopsis (Okushima et al. 2007). Indeed, a growing body of evidence suggested that soluble sugars control plant development through modulating auxin action (Le et al. 2010; Sairanen et al. 2013; Barbier et al. 2015; Meitzel et al. 2021; Morales-Herrera et al. 2023; Stitz et al. 2023). For example, elevation of Tre6P levels in embryo of garden pea (*Pisum sativum*) can promote auxin synthesis by upregulating *TRYPTOPHAN AMINOTRANSFERASE RELATED2* (*TAR2*) gene (Meitzel et al. 2021), on the other hand, auxin impacts Tre6P levels by transcriptionally downregulating Tre6P-degradation gene *TPPB* during LR formation in Arabidopsis (Morales-Herrera et al. 2023). Thus, further uncovering the molecular link between auxin and trehalose metabolism pathway during the CR development will deeply expand the understanding of the regulatory framework of CR in white lupin under P deficiency.

### *LaTRE1* impacts the synthesis of organic acid that contributes to CR function

Previous studies showed that *tps1* mutant in *S. cerevisiae* caused the growth arrest of glucose fermentation because of the inhibition of the glycolytic flux (Gancedo and Flores 2004), and short-term induction in Tre6P level stimulated organic and amino acid biosynthesis in Arabidopsis (Figueroa et al. 2016). In our metabolomic profile, overexpression of *LaTRE1* led to the downregulation of glycolysis and TCA cycle intermediates and the upregulation of amino acid biosynthesis (Fig. 7). Organic acids (e.g. citrate and malate) are thought to contribute to the outstanding efficiency of white lupin to mobilize P in soil (Lambers et al. 2006). We found that the amounts of organic acids, especially malate, were increased in CR after ValA treatment and decreased after *LaTRE1*-overexpression (Fig. 3B; Fig. 7), which was consistent with the previous report that *AtTRE1*, activated by ARABIDOPSIS TRANSCRIPTION ACTIVATION FACTOR 1 (ATAF1) transcriptionally and CALCIUM-DEPENDENT PROTEIN KINASE (AtCPK10) post-translationally, inhibits malate synthesis from starch by decreasing trehalose and Tre6P levels to reduce the turgor pressure of guard cells and trigger stomatal closure (Garapati et al. 2015; Le Cong Huyen Bao Phan et al. 2022). Herein, genes involved in malate metabolism and exudation, including *LaACLA-2* and *LaACLB-1* (Langlade et al. 2002), were differentially expressed in *LaTRE1*-overexpression roots (Fig. 7), and *LaACLA-2* exhibited a positive association with *LaTRE1* during the development of CRs (Fig. 6D). These findings suggested a role of *LaTRE1* in the regulation of CR function through modulating genes involved in the production and excretion of organic acids.

In conclusion, ValA treatment to inhibit trehalase activity stimulates the formation of CR with enhanced organic acid production, whereas overexpression of *LaTRE1* leads to reduced lateral rootlet density and organic acid production under P deficiency, implicating an important role of *LaTRE1* in the regulation of CR formation and function with respect to organic acid production. Until now, no stable transformation protocol was successfully reported in white lupin. We tried to obtain stable *LaTRE1*-overexpressing or -silencing transgenics of white lupin; however, calli induced from transgenic roots failed to differentiate into whole plants. In the future, TILLING (Targeting Induced Local Lesions In Genomes) technology for the characterization of *LaTRE1* loss-of-function mutants could be performed to solidly prove the function of *LaTRE1*. In addition, a number of studies suggested that important developmental and growth phenotypes in plants may be attributed to changes in Tre6P levels rather than trehalose. Whether the phenotypic, transcriptomic, and metabolic changes observed in this study are caused by the alterations of trehalose levels, Tre6P levels or *LaTRE1* require further investigation. Overall, our findings could be considered as a starting point for uncovering the role of trehalose metabolism pathway in regulating the formation and function of white lupin CR under P deficiency.

## Materials and methods

### Plant materials and growth conditions

Seeds of white lupin (*L. albus* cv. “Amiga”) were surface sterilized in 70% (v/v) ethanol for 5 min followed by 15% (v/v) NaClO for 30 min, rinsed thoroughly in sterile distilled water and then germinated on filter papers moistened with saturated 1 mm CaSO_4_ solution in dark for 3 d. Seedlings were transferred into P-sufficient nutrient solutions with 0.25 mm KH_2_PO_4_, 0.5 mm Ca(NO_3_)_2_, 1.75 mm K_2_SO_4_, 1.25 mm MgSO_4_, 25 μμ H_3_BO_3_, 1.5 μμ MnSO_4_, 1.5 μμ ZnSO_4_, 0.5 μμ CuSO_4_, 25 nm (NH_4_)_6_Mo_7_O_24_, and 20 μμ Fe(III)-EDTA at pH 5.8. Replacement of the nutrient solution was carried out every 3 d. Plants were grown in a growth chamber with a day/night cycle of 16 h/8 h at 25°C/23°C. For ValA treatment, 1-wk pre-cultured P-sufficient plants were transferred to the P-sufficient or -deficient (without 0.25 mm KH_2_PO_4_) solutions with different concentrations of ValA (Sigma-Aldrich, USA). Number of CR, F.W. of shoot and root, and the root-to-shoot fresh biomass ratio were analyzed after 3 wk of cultivation.

### Analysis of trehalose metabolism genes in white lupin genome

To identify white lupin *TPS*, *TPP*, and *TRE* candidates, the whole white lupin protein sequences were downloaded from the NCBI database under accession PRJNA592024 and the White Lupin genome portal (www.whitelupin.fr), and a BLASTP-algorithm based search was conducted with an *E*-value ≤ 1e^−10^ threshold by using Arabidopsis (*A. thaliana*) TPS1 (AT1G78580), TPPA (AT5G51460), and TRE1 (AT4G24040) protein sequences as queries. The output putative white lupin TPS, TPP, and TRE protein sequences were submitted to Pfam database (http://pfam.xfam.org/) to confirm the conserved domains, including Glyco_transf_20 (PF00982), Trehalose_PPase (PF02358), and Trehalase (PF01204). Candidate proteins lacking the conserved domains were excluded, and the incorrectly predicted genes were manually curated. Finally, the non-redundant and high-confidence genes were assigned as *LaTPSs*, *LaTPPs*, and *LaTREs* and named on the basis of their positions on chromosomes. Protein sequences of narrow-leafed lupin (*L. angustifolius*), barrel medic (*M. truncatula*), soybean (*G. max*), and common bean (*P. vulgaris*) were downloaded from the Ensembl Plants database, and TRE candidates in these species were identified with the same method. To determine the selection pressure of *TRE1* genes in these 6 plant species mentioned above, the ratios of synonymous substitutions (Ks) and non-synonymous (Ka) nucleotide substitutions (Ka/Ks) of gene pairs were calculated via Simple Ka/Ks Calculator module in TBtools (Chen et al. 2020). The 3-dimensional protein structural model of TRE1 was predicted by AlphaFold (Jumper et al. 2021). Based on MEGA X (v10.0.5) software, multiple sequence alignments were performed using the MUSCLE program (default settings) with full-length TPS, TPP, or TRE protein sequences, and unrooted phylogenetic trees were constructed using the Maximum Likelihood method with 1,000 bootstrap re-samplings.

### RNA extraction and RT-qPCR

Total RNA was extracted from about 1.0 g samples using TRIzol reagent (Invitrogen, USA) in accordance with the manufacturer's instructions. The TransScript One-Step gDNA Removal and cDNA Synthesis SuperMix Kit (TransGen, China) was used for cDNA synthesis, and AceQ qPCR SYBR Green Master Mix (Vazyme, China) was used for amplification with the Bio-Rad CFX96 real-time PCR detection system using gene-specific primers (listed in Supplementary Table S9). The expression level of the gene was calculated using the 2^−ΔCt^ or 2^−ΔΔCt^ method. *LaACTIN* (*Lalb_Chr04g0257761*) was used as an internal control.

### Trehalase activity assay

To determine the effect of ValA on the activity of trehalase in white lupin roots, 1-wk pre-cultured P-sufficient plants were transferred to the P-sufficient or -deficient solutions with or without 200 *μ*M ValA. The roots were collected for the measurement of the trehalase activity by using a Trehalase (THL) Activity Assay Kit (Boxbio, China) according to the manufacturer's instructions. Briefly, root tissues were ground in liquid nitrogen, and the total proteins were extracted in 1 mL 100 mm MES buffer (pH 6.3) containing 1 mm phenylmethylsulfonyl fluoride, 2 mm EDTA, and 5 mg polyvinylpyrrolidone. The supernatant was collected after 12,000 × *g* centrifugation for 10 min twice and then used for protein quantitation and trehalase activity assay. The protein concentration was determined by the Bradford method, and the trehalase activity was measured by estimating the glucose produced by the hydrolysis of trehalose, using the glucose oxidase-peroxidase method with the test kit mentioned. About 0.1 g root tissues were collected and one technical replicate was conducted for one biological replicate and 3 biological replicates were performed. The trehalase enzymatic activity was expressed as the production of μg glucose per mg of protein per minute.

### Measurement of sugar and organic acid

Different types of root tissues cultured in P-sufficient or -deficient nutrient solutions with or without 200 *μ*M ValA treatment for 3 wk were collected and homogenized in 80% (v/v) methanol containing 2 *μ*g/mL of succinic acid-^13^C4 and 25 *μ*g/mL U-^13^C6-glucose using a Tissuelyser (JX-24) with beads for 4 min at 40 Hz. The extraction was kept at −40°C overnight. The supernatant was collected after 15,000 × *g* centrifugation for 15 min and dried with a flow of nitrogen. The residue was dissolved with 50 *μ*L of 50% (v/v) aqueous acetonitrile. Quantification of citrate and isocitrate acids was performed on an Agilent 7890A gas chromatography system coupled to an Agilent 5975C inert MSD system, and quantification of other carbohydrate metabolites was performed on an Agilent 1290 Infinity II UPLC system coupled to a 6470A Triple Quadrupole mass spectrometry. The peak area of the analyte in the sample was compared to the internal standard (succinic acid-^13^C4 or U-^13^C6-glucose), and the concentration was calculated using the calibration curve equation. About 0.5 g different types of root tissues were collected and 1 technical replicate was conducted for 1 biological replicate, and 3 biological replicates were performed. Eight carbohydrate metabolites, including trehalose, glucose, sucrose fumarate, malate, succinate, citrate, and isocitrate were detected and quantified, and Tre6P was undetected from all samples.

### Vector construction

The coding sequence of GFP was amplified using GFP-OE-F/R primers and cloned into pFGC5941 (GeneBank accession no. AY310901) digested by NcoI and BamHI for the construction of *35S::GFP*, and the coding sequence of *LaTRE1* amplified with LaTRE1-OE-F/R primers and cloned into pFGC5941-RFP (the *bar* gene in pFGC5941 was replaced by RFP, modified by our lab) digested by NcoI and BamHI to generate *35S::LaTRE1*. The pTRV1 (pYL192) and pTRV2 (pYL156) vectors have been described previously (Liu et al. 2002), about 300 bp fragments of *LaTRE1* and *LaLBD16* coding sequences were amplified and cloned into pTRV2 digested by EcoRI and BamHI to generate pTRV2*-LaTRE1* and pTRV2*-LaLBD16*. Primers were listed in Supplementary Table S9.

### *A*. *rhizogenes*-mediated hairy root transformation

The *A. rhizogenes*-mediated hairy-root transformation was performed as previously described, with small modifications (Uhde-Stone et al. 2005). Briefly, white lupin seeds were surface sterilized and germinated as aforementioned. When the radicles grew to ∼10 mm, about 3 mm tip sections were removed with a sterile scalpel. The remaining radicles were inoculated with the *A. rhizogenes* strain K599 carrying *35S::GFP* or *35S::LaTRE1* constructs and co-cultivated on Murashige and Skoog (MS, Caisson Labs, USA) plates with 150 *μ*M acetosyringone for 3 d. Seedlings were then transplanted to a pot containing P-sufficient solutions and grown for 10 d. Positive transgenic roots were determined by GFP or RFP fluorescent signals, which were detected using a Leica M205 FA fluorescence stereomicroscope with a 488 nm excitation laser and 525/50 nm emission filter for GFP (Intensity: 55%, Gain: 700) and a 558 nm excitation laser and 624/40 nm emission filter for RFP (Intensity: 55%, Gain: 600). Overexpression of *LaTRE1* in positive transgenic roots was further confirmed by RT-qPCR. Composite plants with *LaTRE1*-overproducing transgenic roots were grown under P deficiency for another 3 wk. Then, the P-starved transgenic roots were used for transcriptome, metabolome and indole-3-acetic acid (IAA) quantification analyses. Ten independent transgenic roots were collected (∼1 g) and 1 technical replicate was conducted for 1 biological replicate and 3 biological replicates were conducted for each analysis.

### Virus-induced gene silencing (VIGS)

VIGS experiment was performed as previously described (Aslam et al. 2022). First, *A. tumefaciens* strain GV3101 carrying pTRV2, pTRV2*-LaTRE1*, pTRV2*-LaLBD16*, or pTRV1, were cultured in LB liquid medium with appropriate antibiotics at 28°C for 16 h. Agrobacterium cells were then collected after 5000 × *g* centrifugation for 5 min and resuspended in infiltration buffer (10 mm MES, 10 mm MgCl_2_, and 200 *μ*M acetosyringone, pH 5.6) to a final OD_600_ = 0.8. The suspensions of pTRV1 and pTRV2*-LaTRE1*, pTRV1 and pTRV2*-LaLBD16*, and pTRV1 and pTRV2 were mixed in a 1:1 (v/v) ratio and infiltrated into roots of 1-wk-old white lupin seedlings grown under P deficiency (3 times in a 3-d interval). After the last inoculation, the plants were grown under P-deficient conditions for another 5 wk in a growth chamber with a day/night cycle of 16 h/8 h at 25°C/23°C. Then, RT-qPCR was performed to determine the silencing efficiency, and phenotypes of the successfully silenced plants were analyzed, including the number of CR, root and shoot F.W., and the root-to-shoot F.W. ratio. At least 3 silenced plants of each VIGS experiment were analyzed.

### Metabolome analysis

Sample preparation, extract analysis, metabolite identification, and quantification were performed at Wuhan MetWare Biotechnology Co., Ltd. (Wuhan, China) following the previously described procedures (Yuan et al. 2018). Data matrices with an intensity of the metabolite features were uploaded to the Analyst 1.6.3 software (AB SCIEX) for statistical analysis. Qualitative analysis of metabolites was conducted according to the secondary spectrum information in the database curated by MetWare Biotechnology Co., Ltd. The relative content of metabolite was calculated according to the corrected mass spectrum peak area. The supervised multivariate method, partial least squares-discriminant analysis, was used to maximize the metabolome differences, and the corresponding VIP was calculated. DAMs were identified with the significance thresholds of FC ≥ 1.3 or ≤ 1/1.3, VIP ≥ 1, and *P* < 0.05.

### Transcriptome analysis

Total RNA extraction, cDNA library construction, and sequencing were performed at Wuhan MetWare Biotechnology Co., Ltd. (Wuhan, China) using an Illumina HiSeq 4000 platform. Paired-end reads 150 bp in length were generated. Cleaned reads were mapped to genes of white lupin using HISAT2 (v2.2.1). Expression levels were calculated as the number of fragments per kilobase of transcript per million mapped reads (FPKM). DEGs were identified using DEseqR package with the significance thresholds of |log_2_FC| ≥ 1, *P* < 0.05 and FDR < 0.2. Mapman annotation and classification of the DEGs were performed using Mercator4 (v2.0). Spearman's correlation analysis was performed in R (v4.0.5) to compare the expression patterns between *LaTRE1* and its regulated DEGs during the development of CR according to the transcriptome data that covers 8 developmental stages of CR (Hufnagel et al. 2020).

### IAA quantification

Samples were harvested and homogenized in a precooled methanol/water/formic acid (15:4:1, v/v/v). The combined extracts were dried with a flow of nitrogen, redissolved in 80% methanol (v/v), and filtered through 0.22 *μ*m filter. Then, sample extracts were analyzed using a UPLC-ESI-MS/MS system (UPLC, ExionLC^TM^Ad; MS, Applied Biosystems 6500 Triple Quadrupole). The concentration of IAA was determined using the external standard method and was expressed as ng/g F.W.

### Statistical analysis

All the data were analyzed with the analysis of variation according to SPSS software (v.24.0), and significant differences were determined by a one-way ANOVA test or unpaired 2-sided Student's *t*-test at *P* < 0.05.

### Accession numbers

The raw reads of the transcriptome analysis were deposited to the NCBI BioProject database under accession number PRJNA973852. Accession numbers of genes in this study are provided in Supplementary Tables S1 and S5 to S8.

## Supplementary Material

kiae290_Supplementary_Data

## Data Availability

Data supporting the findings of this work are available within the paper and its supplemental figures and tables. The raw reads of the transcriptome analysis were deposited to the NCBI BioProject database under accession number PRJNA973852. The datasets generated and analysed in the current study are available from the corresponding author on reasonable request.

## Supplementary data

The following materials are available in the online version of this article.

**Supplementary Figure S1**. Phylogenetic analysis of *TPS* and *TPP* genes in white lupin and Arabidopsis.

**Supplementary Figure S2**. Multiple sequence alignments of TRE1 proteins.

**Supplementary Figure S3**. Alphafold-predicted structures of TRE1 proteins.

**Supplementary Figure S4**. Effect of ValA treatment on trehalase activity in roots of white lupin grown under P-sufficient (+P) and -deficient (−P) conditions.

**Supplementary Figure S5**. Relative levels of key differentially regulated genes and metabolites in *35S::GFP* and *35S::LaTRE1* transgenic roots.

**Supplementary Figure S6**. Expression levels of *LaTRE1* and *LaLBD16* in *LaTRE1*-silenced roots.

**Supplementary Figure S7**. Expression levels of *LaLBD16* and *LaTRE1* in *LaLBD16*-silenced roots.

**Supplementary Figure S8**. Effect of *LaTRE1*-overexpression on auxin action.

**Supplementary Table S1**. Detailed information about the trehalose metabolism genes in white lupin.

**Supplementary Table S2**. Contents of sugars and organic acids in different types of root tissues with or without ValA treatment.

**Supplementary Table S3.** Metabolomic profile of *35S::LaTRE1* and *35S::GFP* transgenic roots under P deficiency.

**Supplementary Table S4.** KEGG enrichment analysis of the DAMs between *35S::LaTRE1* and *35S::GFP* transgenic roots under P deficiency.

**Supplementary Table S5.** The DEGs between *35S::LaTRE1* and *35S::GFP* transgenic roots under P deficiency.

**Supplementary Table S6.** Mapman annotation and classification of the DEGs.

**Supplementary Table S7.** Expression levels of key DEGs related to lateral root formation, organic acid production and excretion.

**Supplementary Table S8.** Spearman's correlation analysis between *LaTRE1* and DEGs during the development of CR.

**Supplementary Table S9.** Primers used in this study.

## Dive Curated Terms

The following phenotypic, genotypic, and functional terms are of significance to the work described in this paper:

ATAF1 Gramene: AT1G01720
ATAF1 Araport: AT1G01720

LBD29 Gramene: AT3G58190
LBD29 Araport: AT3G58190

protein CHEBI: CHEBI:36080

ACL Gramene: At1g10670; At1g60810; At1g09430; At3g06650; At5g49460
ACL Araport: At1g10670; At1g60810; At1g09430; At3g06650; At5g49460

ACLA-2 Gramene: Os11g0696200
ACLA-2 Araport: Os11g0696200

LBD16 Gramene: AT2G42430
LBD16 Araport: AT2G42430

ACLB-1 Gramene: Os01g0300200
ACLB-1 Araport: Os01g0300200

ABA CHEBI: CHEBI:2365

## References

[kiae290-B1] Aslam MM , Pueyo JJ, Pang J, Yang J, Chen W, Chen H, Waseem M, Li Y, Zhang J, Xu W. Root acid phosphatases and rhizobacteria synergistically enhance white lupin and rice phosphorus acquisition. Plant Physiol. 2022:190(4):2449–2465. 10.1093/plphys/kiac418 36066452 PMC9706455

[kiae290-B2] Avonce N , Leyman B, Mascorro-Gallardo JO, Van Dijck P, Thevelein JM, Iturriaga G. The Arabidopsis trehalose-6-P synthase *AtTPS1* gene is a regulator of glucose, abscisic acid, and stress signaling. Plant Physiol. 2004:136(3):3649–3659. 10.1104/pp.104.052084 15516499 PMC527163

[kiae290-B3] Barbier F , Péron T, Lecerf M, Perez-Garcia M-D, Barrière Q, Rolčík J, Boutet-Mercey S, Citerne S, Lemoine R, Porcheron B, et al Sucrose is an early modulator of the key hormonal mechanisms controlling bud outgrowth in *Rosa hybrida*. J Exp Bot. 2015:66(9):2569–2582. 10.1093/jxb/erv047 25873679 PMC4986866

[kiae290-B4] Barraza A , Estrada-Navarrete G, Rodriguez-Alegria ME, Lopez-Munguia A, Merino E, Quinto C, Sanchez F. Down-regulation of *PvTRE1* enhances nodule biomass and bacteroid number in the common bean. New Phytol. 2013:197(1):194–206. 10.1111/nph.12002 23121215

[kiae290-B5] Brundrett MC . Coevolution of roots and mycorrhizas of land plants. New Phytol. 2002:154(2):275–304. 10.1046/j.1469-8137.2002.00397.x 33873429

[kiae290-B6] Chen C , Chen H, Zhang Y, Thomas HR, Frank MH, He Y, Xia R. TBtools: an integrative toolkit developed for interactive analyses of big biological data. Mol Plant. 2020:13(8):1194–1202. 10.1016/j.molp.2020.06.009 32585190

[kiae290-B7] Cheng L , Tang X, Vance CP, White PJ, Zhang F, Shen J. Interactions between light intensity and phosphorus nutrition affect the phosphate-mining capacity of white lupin (*Lupinus albus* L.). J Exp Bot. 2014:65(12):2995–3003. 10.1093/jxb/eru135 24723402 PMC4071820

[kiae290-B8] Chilton M-D , Tepfer DA, Petit A, David C, Casse-Delbart F, Tempé J. *Agrobacterium rhizogenes* inserts T-DNA into the genomes of the host plant root cells. Nature. 1982:295(5848):432–434. 10.1038/295432a0

[kiae290-B9] Collier R , Fuchs B, Walter N, Kevin Lutke W, Taylor CG. *Ex vitro* composite plants: an inexpensive, rapid method for root biology. Plant J. 2005:43(3):449–457. 10.1111/j.1365-313X.2005.02454.x 16045479

[kiae290-B10] Durrett TP , Gassmann W, Rogers EE. The FRD3-mediated efflux of citrate into the root vasculature is necessary for efficient iron translocation. Plant Physiol. 2007:144(1):197–205. 10.1104/pp.107.097162 17351051 PMC1913786

[kiae290-B11] Eastmond PJ , Astley HM, Parsley K, Aubry S, Williams BP, Menard GN, Craddock CP, Nunes-Nesi A, Fernie AR, Hibberd JM. Arabidopsis uses two gluconeogenic gateways for organic acids to fuel seedling establishment. Nat Commun. 2015:6(1):6659–6659. 10.1038/ncomms7659 25858700 PMC4403315

[kiae290-B12] Fatland BL , Nikolau BJ, Wurtele ES. Reverse genetic characterization of cytosolic acetyl-CoA generation by ATP-citrate lyase in Arabidopsis. Plant Cell. 2005:17(1):182–203. 10.1105/tpc.104.026211 15608338 PMC544498

[kiae290-B13] Figueroa CM , Feil R, Ishihara H, Watanabe M, Kölling K, Krause U, Höhne M, Encke B, Plaxton WC, Zeeman SC. Trehalose 6–phosphate coordinates organic and amino acid metabolism with carbon availability. Plant J. 2016:85(3):410–423. 10.1111/tpj.13114 26714615

[kiae290-B14] Frison M , Parrou JL, Guillaumot D, Masquelier D, François J, Chaumont F, Batoko H. The *Arabidopsis thaliana* trehalase is a plasma membrane-bound enzyme with extracellular activity. FEBS Lett. 2007:581(21):4010–4016. 10.1016/j.febslet.2007.07.036 17673210

[kiae290-B15] Gancedo C , Flores C. The importance of a functional trehalose biosynthetic pathway for the life of yeasts and fungi. FEMS Yeast Res. 2004:4(4–5):351–359. 10.1016/S1567-1356(03)00222-8 14734015

[kiae290-B16] Garapati P , Feil R, Lunn JE, Van Dijck P, Balazadeh S, Mueller-Roeber B. Transcription factor Arabidopsis Activating Factor1 integrates carbon starvation responses with trehalose metabolism. Plant Physiol. 2015:169(1):379. 10.1104/pp.15.00917 26149570 PMC4577426

[kiae290-B17] Goddijn OJ , Verwoerd TC, Voogd E, Krutwagen RW, de Graaf PT, van Dun K, Poels J, Ponstein AS, Damm B, Pen J. Inhibition of trehalase activity enhances trehalose accumulation in transgenic plants. Plant Physiol. 1997:113(1):181–190. 10.1104/pp.113.1.181 9008394 PMC158129

[kiae290-B18] Goh T , Kasahara H, Mimura T, Kamiya Y, Fukaki H. Multiple AUX/IAA-ARF modules regulate lateral root formation: the role of *Arabidopsis* SHY2/IAA3-mediated auxin signalling. Philos Trans R Soc B Biol Sci. 2012:367(1595):1461–1468. 10.1098/rstb.2011.0232 PMC3321683 22527388

[kiae290-B19] Gómez LD , Gilday A, Feil R, Lunn JE, Graham IA. AtTPS1-mediated trehalose 6-phosphate synthesis is essential for embryogenic and vegetative growth and responsiveness to ABA in germinating seeds and stomatal guard cells. Plant J. 2010:64(1):1–13. 10.1111/j.1365-313X.2010.04312.x 20659274

[kiae290-B20] Herrmann KM , Weaver LM. The shikimate pathway. Annu Rev Plant Physiol Plant Mol Biol. 1999:50(1):473–503. 10.1146/annurev.arplant.50.1.473 15012217

[kiae290-B21] Hoekenga OA , Maron LG, Piñeros MA, Cançado GMA, Shaff J, Kobayashi Y, Ryan PR, Dong B, Delhaize E, Sasaki T, et al *AtALMT1*, which encodes a malate transporter, is identified as one of several genes critical for aluminum tolerance in *Arabidopsis*. Proc Natl Acad Sci USA. 2006:103(25):9738–9743. 10.1073/pnas.0602868103 16740662 PMC1480476

[kiae290-B22] Hufnagel B , Marques A, Soriano A, Marquès L, Divol F, Doumas P, Sallet E, Mancinotti D, Carrere S, Marande W, et al High-quality genome sequence of white lupin provides insight into soil exploration and seed quality. Nat Commun. 2020:11(1):492. 10.1038/s41467-019-14197-9 31980615 PMC6981116

[kiae290-B23] Jumper J , Evans R, Pritzel A, Green T, Figurnov M, Ronneberger O, Tunyasuvunakool K, Bates R, Žídek A, Potapenko A, et al Highly accurate protein structure prediction with AlphaFold. Nature. 2021:596(7873):583–589. 10.1038/s41586-021-03819-2 34265844 PMC8371605

[kiae290-B24] Lambers H , Shane MW, Cramer MD, Pearse SJ, Veneklaas EJ. Root structure and functioning for efficient acquisition of phosphorus: matching morphological and physiological traits. Ann Bot. 2006:98(4):693–713. 10.1093/aob/mcl114 16769731 PMC2806175

[kiae290-B25] Langlade NB , Messerli G, Weisskopf L, Plaza S, Tomasi N, Smutny J, Neumann G, Martinoia E, Massonneau A. ATP citrate lyase: cloning, heterologous expression and possible implication in root organic acid metabolism and excretion. Plant Cell Environ. 2002:25(11):1561–1569. 10.1046/j.1365-3040.2002.00936.x

[kiae290-B26] Le CS , Schmelz EA, Chourey PS. Sugar levels regulate tryptophan-dependent auxin biosynthesis in developing maize kernels. Plant Physiol. 2010:153(1):306–318. 10.1104/pp.110.155226 20237017 PMC2862422

[kiae290-B27] Le Cong Huyen Bao Phan T , Crepin N, Rolland F, Van Dijck P. Two trehalase isoforms, produced from a single transcript, regulate drought stress tolerance in *Arabidopsis thaliana*. Plant Mol Biol. 2022:108(6):531–547. 10.1007/s11103-022-01243-2 35088230

[kiae290-B28] Li L , Sheen J. Dynamic and diverse sugar signaling. Curr Opin Plant Biol. 2016:33:116–125. 10.1016/j.pbi.2016.06.018 27423125 PMC5050104

[kiae290-B29] Liu Y , Schiff M, Marathe R, Dinesh-Kumar SP. Tobacco *Rar1, EDS1* and *NPR1/NIM1* like genes are required for *N*-mediated resistance to tobacco mosaic virus. Plant J. 2002:30(4):415–429. 10.1046/j.1365-313X.2002.01297.x 12028572

[kiae290-B30] López M , Tejera NA, Iribarne C, Lluch C, Herrera-Cervera JA. Trehalose and trehalase in root nodules of *Medicago truncatula* and *Phaseolus vulgaris* in response to salt stress. Physiol Plant. 2008:134(4):575–582. 10.1111/j.1399-3054.2008.01162.x 18823327

[kiae290-B31] López M , Tejera NA, Lluch C. Validamycin A improves the response of *Medicago truncatula* plants to salt stress by inducing trehalose accumulation in the root nodules. J Plant Physiol. 2009:166(11):1218–1222. 10.1016/j.jplph.2008.12.011 19232773

[kiae290-B32] Lunn JE . Gene families and evolution of trehalose metabolism in plants. Funct Plant Biol. 2007:34(6):550–563. 10.1071/FP06315 32689383

[kiae290-B33] Lunn JE , Delorge I, Figueroa CM, Van Dijck P, Stitt M. Trehalose metabolism in plants. Plant J. 2014:79(4):544–567. 10.1111/tpj.12509 24645920

[kiae290-B34] Meitzel T , Radchuk R, McAdam EL, Thormählen I, Feil R, Munz E, Hilo A, Geigenberger P, Ross JJ, Lunn JE, et al Trehalose 6-phosphate promotes seed filling by activating auxin biosynthesis. New Phytol. 2021:229(3):1553–1565. 10.1111/nph.16956 32984971

[kiae290-B35] Meng ZB , Chen LQ, Suo D, Li GX, Tang CX, Zheng SJ. Nitric oxide is the shared signalling molecule in phosphorus- and iron-deficiency-induced formation of cluster roots in white lupin (*Lupinus albus*). Ann Bot. 2012:109(6):1055–1064. 10.1093/aob/mcs024 22351487 PMC3336943

[kiae290-B36] Miller SS , Liu J, Allan DL, Menzhuber CJ, Fedorova M, Vance CP. Molecular control of acid phosphatase secretion into the rhizosphere of proteoid roots from phosphorus-stressed white lupin. Plant Physiol. 2001:127(2):594. 10.1104/pp.010097 11598233 PMC125094

[kiae290-B37] Morales-Herrera S , Jourquin J, Coppé F, Lopez-Galvis L, De Smet T, Safi A, Njo M, Griffiths CA, Sidda JD, Mccullagh JSO, et al Trehalose-6-phosphate signaling regulates lateral root formation in *Arabidopsis thaliana*. Proc Natl Acad Sci USA. 2023:120(40):e2302996120. 10.1073/pnas.2302996120 PMC10556606 37748053

[kiae290-B38] Müller J , Aeschbacher RA, Wingler A, Boller T, Wiemken A. Trehalose and trehalase in Arabidopsis. Plant Physiol. 2001:125(2):1086–1093. 10.1104/pp.125.2.1086 11161063 PMC64907

[kiae290-B39] Müller J , Gödde V, Niehaus K, Zörb C. Metabolic adaptations of white lupin roots and shoots under phosphorus deficiency. Front Plant Sci. 2015:6:1014. 10.3389/fpls.2015.01014 26635840 PMC4656794

[kiae290-B40] Neumann G , Martinoia E. Cluster roots—an underground adaptation for survival in extreme environments. Trends Plant Sci. 2002:7(4):162–167. 10.1016/S1360-1385(02)02241-0 11950612

[kiae290-B41] Neumann G , Massonneau A, Martinoia E, Römheld V. Physiological adaptations to phosphorus deficiency during proteoid root development in white lupin. Planta. 1999:208(3):373–382. 10.1007/s004250050572

[kiae290-B42] Okushima Y , Fukaki H, Onoda M, Theologis A, Tasaka M. ARF7 and ARF19 regulate lateral root formation via direct activation of *LBD/ASL* genes in *Arabidopsis*. Plant Cell. 2007:19(1):118–130. 10.1105/tpc.106.047761 17259263 PMC1820965

[kiae290-B43] O’Rourke JA , Yang SS, Miller SS, Bucciarelli B, Liu J, Rydeen A, Bozsoki Z, Uhde-Stone C, Tu ZJ, Allan D, et al An RNA-Seq transcriptome analysis of orthophosphate-deficient white lupin reveals novel insights into phosphorus acclimation in plants. Plant Physiol. 2013:161(2):705. 10.1104/pp.112.209254 23197803 PMC3561014

[kiae290-B44] Paul MJ , Primavesi LF, Jhurreea D, Zhang Y. Trehalose metabolism and signaling. Annu Rev Plant Biol. 2008:59(1):417–441. 10.1146/annurev.arplant.59.032607.092945 18257709

[kiae290-B45] Penfield S , Clements S, Bailey KJ, Gilday AD, Leegood RC, Gray JE, Graham IA. Expression and manipulation of *PHOSPHOENOLPYRUVATE CARBOXYKINASE 1* identifies a role for malate metabolism in stomatal closure. Plant J. 2012:69(4):679–688. 10.1111/j.1365-313X.2011.04822.x 22007864

[kiae290-B46] Ponnu J , Schlereth A, Zacharaki V, Działo MA, Abel C, Feil R, Schmid M, Wahl V. The trehalose 6-phosphate pathway impacts vegetative phase change in *Arabidopsis thaliana*. Plant J. 2020:104(3):768–780. 10.1111/tpj.14965 32799402

[kiae290-B47] Rodriguez-Medina C , Atkins CA, Mann AJ, Jordan ME, Smith PMC. Macromolecular composition of phloem exudate from white lupin (*Lupinus albus* L.). BMC Plant Biol. 2011:11(1):36. 10.1186/1471-2229-11-36 21342527 PMC3055823

[kiae290-B48] Romero C , Bellés JM, Vayá JL, Serrano R, Culiáñez-Macià FA. Expression of the yeast trehalose-6-phosphate synthase gene in transgenic tobacco plants: pleiotropic phenotypes include drought tolerance. Planta. 1997:201(3):293–297. 10.1007/s004250050069 19343407

[kiae290-B49] Ruan Y-L . Sucrose metabolism: gateway to diverse carbon use and sugar signaling. Annu Rev Plant Biol. 2014:65(1):33–67. 10.1146/annurev-arplant-050213-040251 24579990

[kiae290-B50] Sairanen I , Novák O, Pěnčík A, Ikeda Y, Jones B, Sandberg G, Ljung K. Soluble carbohydrates regulate auxin biosynthesis via PIF proteins in *Arabidopsis*. Plant Cell. 2013:24(12):4907–4916. 10.1105/tpc.112.104794 PMC3556965 23209113

[kiae290-B51] Schachtman DP , Reid RJ, Ayling SM. Phosphorus uptake by plants: from soil to cell. Plant Physiol. 1998:116(2):447–453. 10.1104/pp.116.2.447 9490752 PMC1539172

[kiae290-B52] Secco D , Shou H, Whelan J, Berkowitz O. RNA-Seq analysis identifies an intricate regulatory network controlling cluster root development in white lupin. BMC Genomics. 2014:15(1):230. 10.1186/1471-2164-15-230 24666749 PMC4028058

[kiae290-B53] Shane MW , Feil R, Lunn JE, Plaxton WC. Light-dependent activation of phosphoenolpyruvate carboxylase by reversible phosphorylation in cluster roots of white lupin plants: diurnal control in response to photosynthate supply. Ann Bot. 2016:118(4):637–643. 27063365 10.1093/aob/mcw040 PMC5055616

[kiae290-B54] Shane MW , Lambers H. Cluster roots: a curiosity in context. Plant Soil. 2005:274(1–2):101–125. 10.1007/s11104-004-2725-7

[kiae290-B55] Shen J , Li H, Neumann G, Zhang F. Nutrient uptake, cluster root formation and exudation of protons and citrate in *Lupinus albus* as affected by localized supply of phosphorus in a split-root system. Plant Sci. 2005:168(3):837–845. 10.1016/j.plantsci.2004.10.017

[kiae290-B56] Stitz M , Kuster D, Reinert M, Schepetilnikov M, Berthet B, Reyes-Hernández J, Janocha D, Artins A, Boix M, Henriques R, et al TOR acts as a metabolic gatekeeper for auxin-dependent lateral root initiation in *Arabidopsis thaliana*. EMBO J. 2023:42(10):e111273. 10.15252/embj.2022111273 37021425 PMC10183831

[kiae290-B57] Sun G , Wase N, Shu S, Jenkins J, Zhou B, Torres-Rodríguez JV, Chen C, Sandor L, Plott C, Yoshinga Y. Genome of *Paspalum vaginatum* and the role of trehalose mediated autophagy in increasing maize biomass. Nat Commun. 2022:13(1):7731. 10.1038/s41467-022-35507-8 36513676 PMC9747981

[kiae290-B58] Sun L , Tian J, Zhang H, Liao H. Phytohormone regulation of root growth triggered by P deficiency or Al toxicity. J Exp Bot. 2016:67(12):3655–3664. 10.1093/jxb/erw188 27190050

[kiae290-B59] Tian L , Peel GJ, Lei Z, Aziz N, Dai X, He J, Watson B, Zhao PX, Sumner LW, Dixon RA. Transcript and proteomic analysis of developing white lupin (*Lupinus albus* L.) roots. BMC Plant Biol. 2009:9(1):1. 10.1186/1471-2229-9-1 19123941 PMC2630931

[kiae290-B60] Uhde-Stone C , Gilbert G, Johnson JM-F, Litjens R, Zinn KE, Temple SJ, Vance CP, Allan DL. Acclimation of white lupin to phosphorus deficiency involves enhanced expression of genes related to organic acid metabolism. Plant Soil. 2003:248(1/2):99–116. 10.1023/A:1022335519879

[kiae290-B61] Uhde-Stone C , Liu J, Zinn KE, Allan DL, Vance CP. Transgenic proteoid roots of white lupin: a vehicle for characterizing and silencing root genes involved in adaptation to P stress. Plant J. 2005:44(5):840–853. 10.1111/j.1365-313X.2005.02573.x 16297074

[kiae290-B62] Van Houtte H , Vandesteene L, López-Galvis L, Lemmens L, Kissel E, Carpentier S, Feil R, Avonce N, Beeckman T, Lunn JE, et al Overexpression of the trehalase gene *AtTRE1* leads to increased drought stress tolerance in Arabidopsis and is involved in abscisic acid-induced stomatal closure. Plant Physiol. 2013:161(3):1158. 10.1104/pp.112.211391 23341362 PMC3585587

[kiae290-B63] Wang W , Chen Q, Xu S, Liu W-C, Zhu X, Song C-P. Trehalose-6-phosphate phosphatase E modulates ABA-controlled root growth and stomatal movement in *Arabidopsis*. J Integr Plant Biol. 2020:62(10):1518–1534. 10.1111/jipb.12925 32167237 PMC7586804

[kiae290-B64] Wang Z , Shen J, Ludewig U, Neumann G. A re-assessment of sucrose signaling involved in cluster-root formation and function in phosphate-deficient white lupin (*Lupinus albus*). Physiol Plant. 2014a:154(3):407–419. 10.1111/ppl.12311 25412792

[kiae290-B65] Wang Z , Straub D, Yang H, Kania A, Shen J, Ludewig U, Neumann G. The regulatory network of cluster-root function and development in phosphate-deficient white lupin (*Lupinus albus*) identified by transcriptome sequencing. Physiol Plant. 2014b:151(3):323–338. 10.1111/ppl.12187 24635386

[kiae290-B66] Watt M , Evans JR. Linking development and determinacy with organic acid efflux from proteoid roots of white lupin grown with low phosphorus and ambient or elevated atmospheric CO_2_ concentration. Plant Physiol. 1999a:120(3):705. 10.1104/pp.120.3.705 10398705 PMC59308

[kiae290-B67] Watt M , Evans JR. Proteoid roots. Physiology and development. Plant Physiol. 1999b:121(2):317. 10.1104/pp.121.2.317 10517822 PMC1539228

[kiae290-B68] Xu W , Zhang Q, Yuan W, Xu F, Muhammad Aslam M, Miao R, Li Y, Wang Q, Li X, Zhang X, et al The genome evolution and low-phosphorus adaptation in white lupin. Nat Commun. 2020:11(1):1069. 10.1038/s41467-020-14891-z 32103018 PMC7044338

[kiae290-B69] Yang Y , Wang L, Zhang D, Che Z, Wang Q, Cui R, Zhao W, Huang F, Zhang H, Cheng H, et al Soybean type-B response regulator GmRR1 mediates phosphorus uptake and yield by modifying root architecture. Plant Physiol. 2024:194(3):1527–1544. 10.1093/plphys/kiad570 37882637

[kiae290-B70] Yuan H , Zeng X, Shi J, Xu Q, Wang Y, Jabu D, Sang Z, Nyima T. Time-course comparative metabolite profiling under osmotic stress in tolerant and sensitive Tibetan hulless barley. Biomed Res Int. 2018:2018:9415409. 10.1155/2018/9415409 30671479 PMC6323448

[kiae290-B71] Zhang Y , Mitsuda N, Yoshizumi T, Horii Y, Oshima Y, Ohme-Takagi M, Matsui M, Kakimoto T. Two types of bHLH transcription factor determine the competence of the pericycle for lateral root initiation. Nat Plants. 2021:7(5):633–643. 10.1038/s41477-021-00919-9 34007039

