# Supplementary material for: The white lupin trehalase gene LaTRE1 regulates cluster root formation and function under phosphorus deficiency
Source: Plant Physiol. 2024 May 28;196(4):2184–98. doi: 10.1093/plphys/kiae290 (PMC11637477; doi:10.1093/plphys/kiae290)
Supplement: kiae290_Supplementary_Data [file kiae290_supplementary_data.zip › PP2024RA00452DR1_Supplemental_Figures_S1_8.pdf]

Supplementary Figures

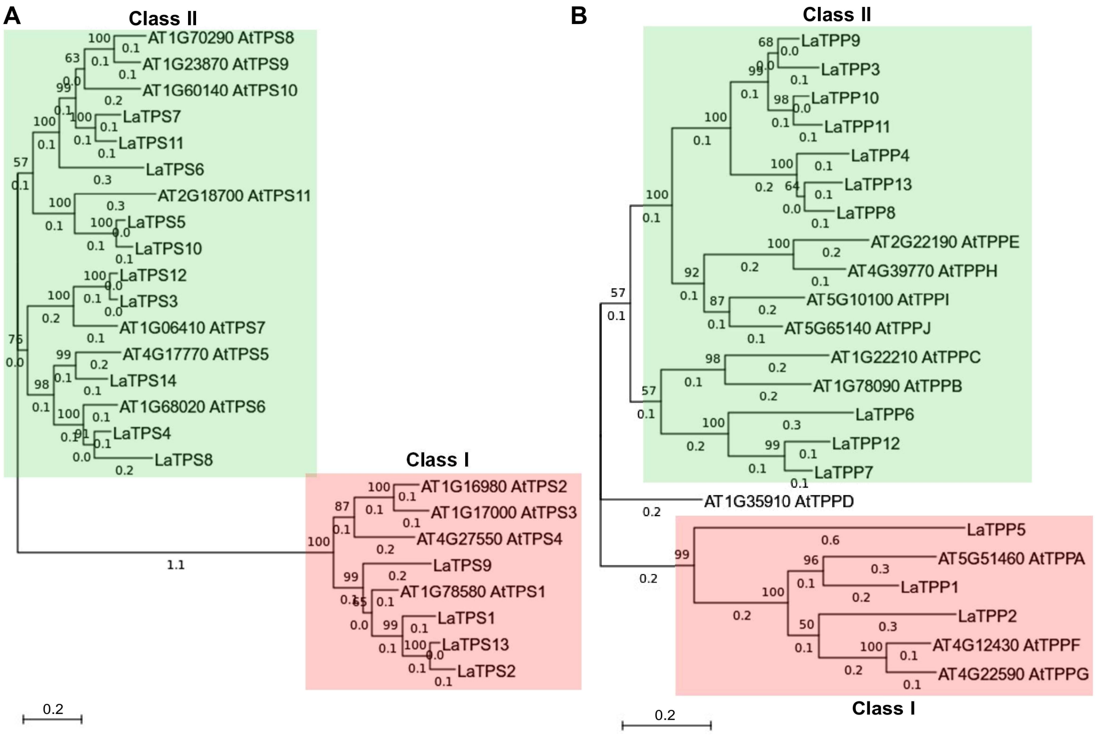

Supplementary Figure S1. Phylogenetic analysis of *TPS* and *TPP* genes in white lupin and Arabidopsis. **A,** Phylogenetic analysis of *TPS* genes. **B,** Phylogenetic analysis of *TPP* genes. Tree scale bar represents distance as the number of substitutions per site. *TPS*, *TREHALOSE-6-PHOSPHATE SYNTHASE*; *TPP*, *TREHALOSE-6-PHOSPHATE PHOSPHATASE*.

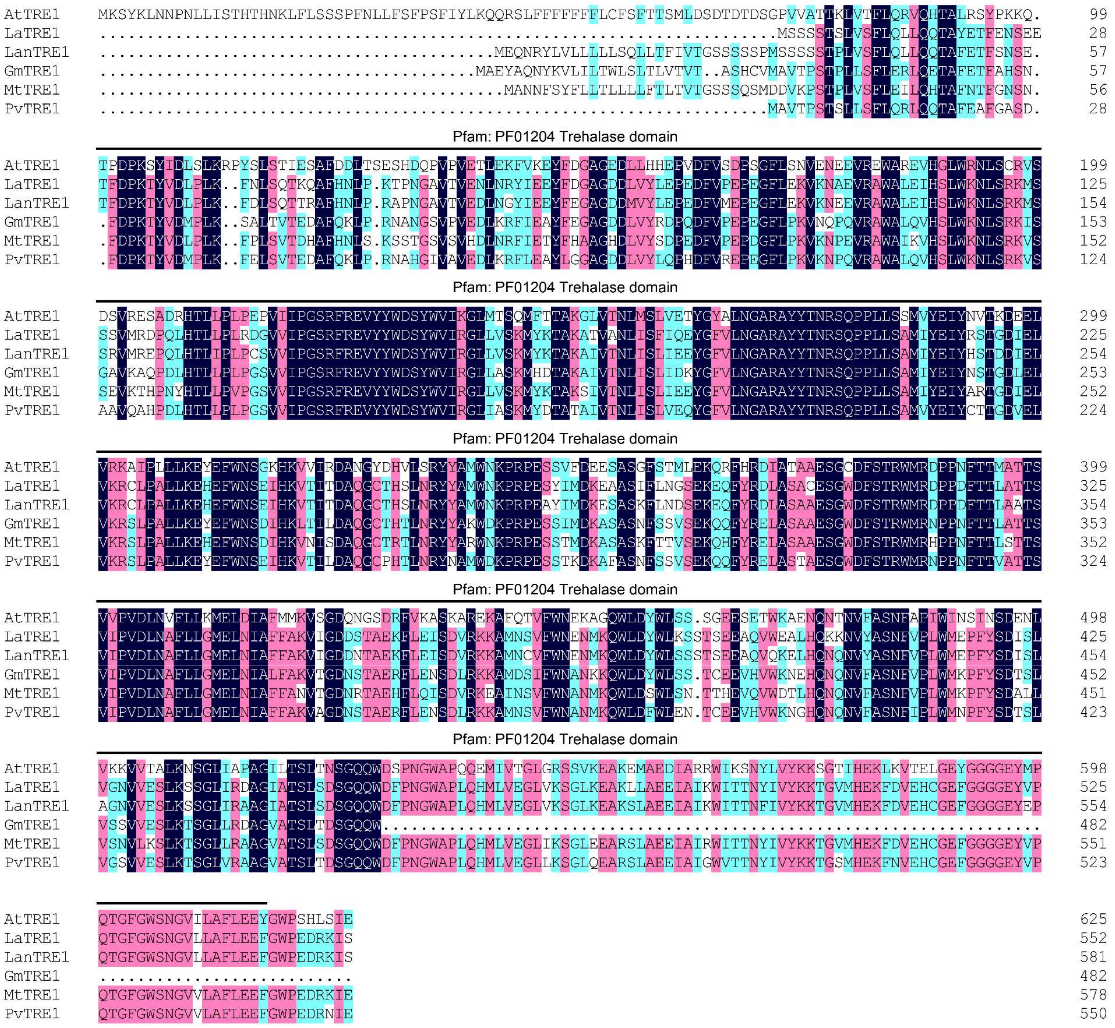

Supplementary Figure S2. Multiple sequence alignments of TRE1 proteins. TRE, TREHALASE; At, *Arabidopsis thaliana*; La, *Lupinus albus*; Lan, *Lupinus angustifolius*; Gm, *Glycine max*; Mt, *Medicago truncatula*; Pv, *Phaseolus vulgaris*. The trehalase domains (Pfam: PF01204) of TRE proteins were indicated.

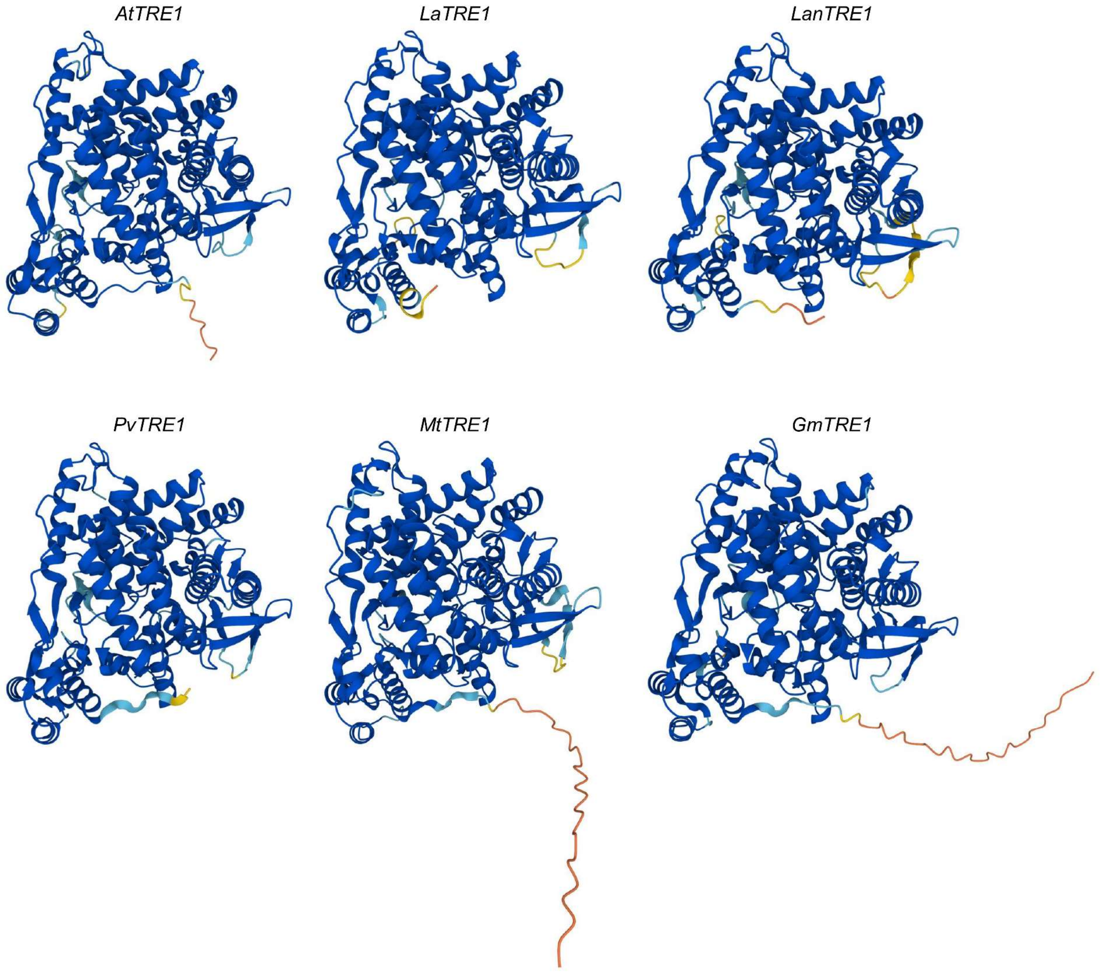

Supplementary Figure S3. Alphafold-predicted structures of TRE1 proteins. At, *Arabidopsis thaliana*; La, *Lupinus albus*; Lan, *Lupinus angustifolius*; Gm, *Glycine max*; Mt, *Medicago truncatula*; Pv, *Phaseolus vulgaris*.

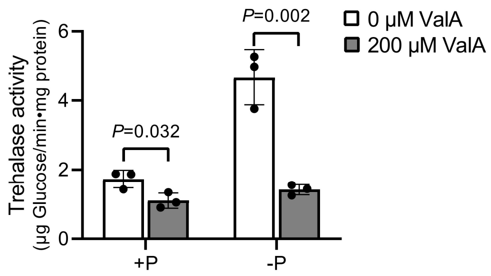

Supplementary Figure S4. Effect of validamycin A (ValA) treatment on trehalase activity in roots of white lupin grown under P-sufficient (+P) and -deficient (-P) conditions. The trehalase activity was expressed as the production of µg glucose per mg of protein per minute. Error bars represent SE, n = 3 biological replicates, *P* value was determined by unpaired two-sided student's *t* test.

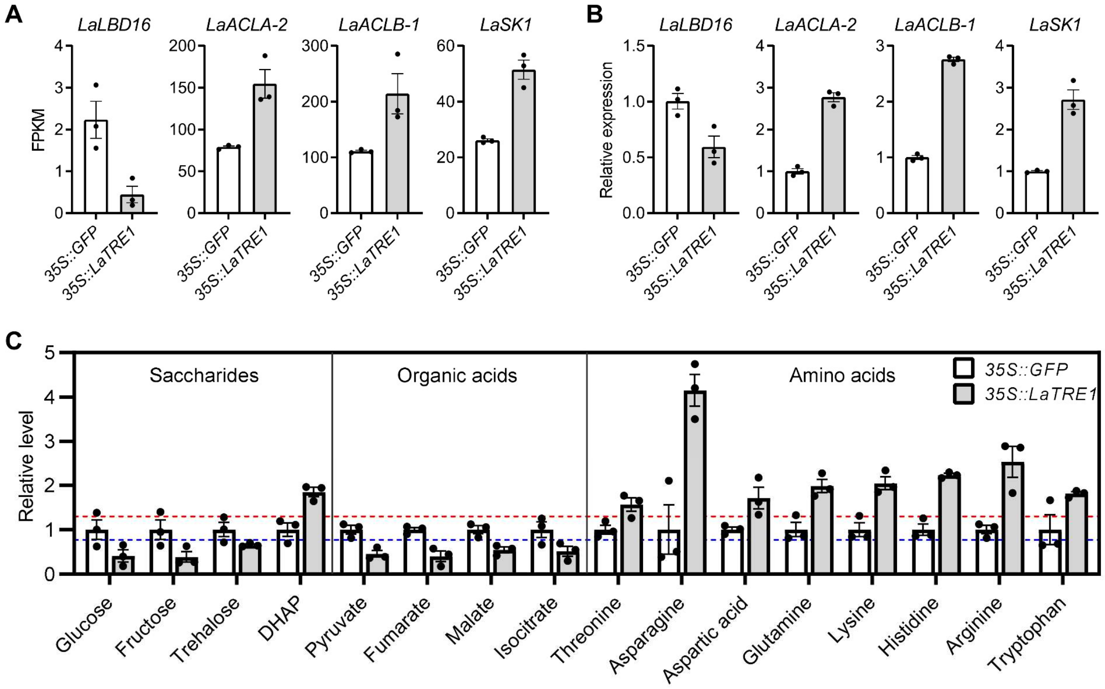

Supplementary Figure S5. Relative levels of key differentially regulated genes and metabolites in *35S::GFP* and *35S::LaTRE1* transgenic roots. **A,** The FPKM values of key differentially regulated genes in our transcriptomic data. **B,** RT-qPCR validation of the expression levels of key genes in (**A**). **C,** The relative levels of key differentially regulated metabolites in our metabolomic profile. The average levels of genes (**B**) and metabolites (**C**) in *35S::GFP* were set as 1. Error bars represent SE, n = 3 biological replicates for (**A**) and (**C**), and independent transgenic roots for (**B**). FPKM in (**A**) represents the fragments per kilobase of transcript per million mapped reads. The red and blue dotted lines in (**C**) represent the thresholds of up- (fold change ≥ 1.3) and down-regulated (fold change ≤ 0.77) metabolites in *35S::LaTRE1* trangenic roots compared to those in *35S::GFP* transgenic roots. DHAP, dihydroxyacetone phosphate.

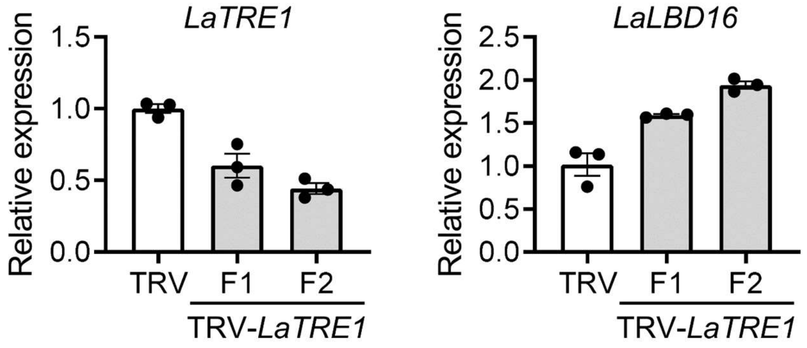

Supplementary Figure S6. Relative expression levels of *LaTRE1* and *LaLBD16* in *LaTRE1*-silenced roots. TRV, empty vector control; TRV-*LaTRE1*-F1 or -F2, two independent *LaTRE1*-silenced lines generated according to different fragments (F1 and F2) of the coding sequence. The average expression levels in controls (TRV) were set as 1. Error bars represent SE, n = 3 individual plants.

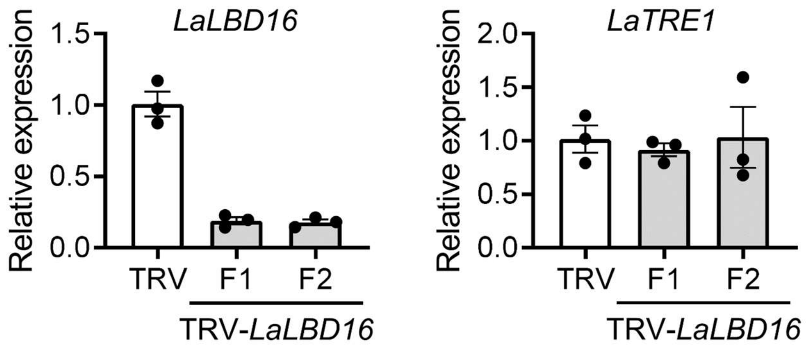

Supplementary Figure S7. Expression levels of *LaLBD16* and *LaTRE1* in *LaLBD16*-silenced roots. TRV, empty vector control; TRV-*LaLBD16*-F1 or -F2, two independent *LaLBD16*-silenced lines generated according to different fragments (F1 and F2) of the coding sequence. The average expression levels in controls (TRV) were set as 1. Error bars represent SE, n = 3 individual plants.

A

Gene ID	Best hit in *Arabidopsis*	Gene symbol
Lalb_Chr20g0111371	AT2G01820	*TMK3*
Lalb_Chr06g0168991	AT1G04240	*SHY2/IAA3*
Lalb_Chr25g0285261	AT1G53700	*WAG1*
Lalb_Chr01g0007121	AT1G56150	*SAUR71*
Lalb_Chr03g0031661	AT1G56150	*SAUR71*
Lalb_Chr19g0133351	AT1G56150	*SAUR71*

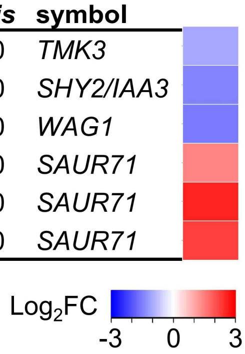

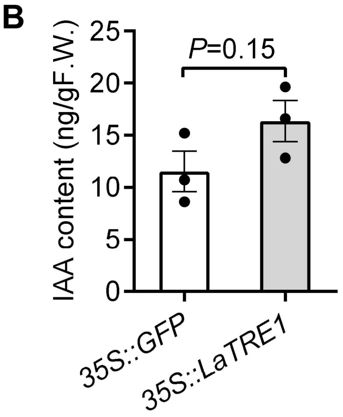

Supplementary Figure S8. Effect of *LaTRE1*-overexpression on auxin action. **A,** The differentially expressed auxin-related genes between *35S::LaTRE1* and *35S::GFP*. FC, fold change. **B,** Contents of IAA in *35S::GFP* and *35S::LaTRE1* transgenic roots. Error bars represent SE, n = 3 biological replicates, *P* value was determined by unpaired two-sided student's *t* test. F.W., fresh weight.
