# Supplementary material for: The white lupin trehalase gene LaTRE1 regulates cluster root formation and function under phosphorus deficiency
Source: Plant Physiol. 2024 May 28;196(4):2184–98. doi: 10.1093/plphys/kiae290 (PMC11637477; doi:10.1093/plphys/kiae290)
Supplement: kiae290_Supplementary_Data [file pp.00452.2024-s04.pdf › pp.00452.2024-s04.pdf]

Plant Physiology®

Trehalase gene LaTRE1 is important for the formation and function of cluster roots in white lupin under P-deficiency

Tianyu Xia, Xiaoqi Zhu, Yujie Zhan, Bowen Liu, Xiangxue Zhou, Qian Zhang, and Weifeng Xu

Review Timeline:

Submission Date:	13-Apr-2023
Editorial Decision:	07-May-2023
Resubmission Received:	28-Mar-2024
Editorial Decision:	11-Apr-2024
Revision Received:	29-Apr-2024
Accepted:	29-Apr-2024

07-May-2023

Prof. Dr. Weifeng Xu
Fujian Agriculture and Forestry University
plant science
Jinshan Fuzhou, orcid|||||||
China

RE: Trehalase gene LaTRE1 is important for the formation and function of cluster roots in white lupin under P-deficiency

Dear Dr. Weifeng Xu:

Thank you for giving us the opportunity to review your manuscript for publication in the Plant Physiology. The reviewers have found the manuscript of interest, but have raised a number of concerns that will require your attention. As these concerns are of some gravity and will entail substantial changes to the manuscript, you are likely to need additional time to address them. I must therefore reject the manuscript in its present form. Nonetheless, if you are able to address the points raised in review, I would welcome your resubmission of a manuscript that incorporates your responses.

In particular, I direct your attention to the following comments for consideration in a resubmitted manuscript.

The authors investigated the phenotype of LaTRE1-overexpression lines, but did not perform RNAi or knockout of this gene in the roots. Although the authors used the trehalase inhibitor ValA, this may not completely demonstrate the phenotype of LaTRE1 suppression. Additional genetic test is necessary.

Is the LaTRE1 gene highly expressed in the roots compared to other tissues (leaves, stems, flowers) in white lupin? This should be clarified.

Considering the instability of instantaneous hairy root transformation, four independent transgenic roots were not sufficient to elucidate this phenotype of LaLBD16 overexpression. Have two independent experiments been conducted (at different times) and shown similar results? Please repeat and confirm these results

Please note that any resubmission will be treated as a new entry to the system and is likely to go out for review.

If you do have any queries or concerns, you are welcome to contact me in the first instance.

Thank you again for allowing Plant Physiology to review your work. We look forward to hearing from you soon.

Sincerely,

Editor, Plant Physiology

-- Reviewer comments:
Reviewer #1 (Comments for the Author):

The manuscript by Xia et al. reported the role of trehalase metabolism in modulating CRs by manipulation the activity of TRE1 in white lupin. In general, this work represents a very interesting topic by combing the CRs development and carbon metabolism. The organization of the manuscript is logical, and presented data is very solid to support the major conclusion. I only have several concerns that needed to be addressed.

1. P deficiency induces the expression of TRE1 and the formation CRs, whereas TRE1 would repress the formation of CRs. How to explain this discrepancy?

2. The authors used ValA to inhibit the activity of trehalase to investigate the role of TRE1. It needs to show the data that the activity of trehalase is really and specially repressed.

3. The data of CRs in the TRE1 over-expression transgenic plants is not shown in this manuscript. Can the authors give some explanations?

4. The authors showed that LBD16 might be an important downstream gene in mediating TRE1-modulated root development. It would be necessary to examine the expression of LBD16 under ValA treatment, which would further support the regulatory role

of TRE1.

Reviewer #2 (Comments for the Author):

In this study, the authors described how a trehalose metabolism gene LaTRE1 affects cluster roots (CRs) formation and function via pharmacological inhibition and A. rhizogenes-mediated overexpression of this gene. Comparative transcriptome and metabolome analyses further advanced in the understanding of LaTRE1 biochemical role. Eventually they found evidence that LaTRE1 impacts the synthesis of organic acids that contribute to CRs function. It is an interesting contribution. Some concerns should be addressed:

1, Lines 105-109, 367-368 and Figures 1B, S1: The phylogenetic tree is not sufficient to clearly separate different clades, because of missing phylogenetic information. Please provide detailed information on the settings in the Materials & Methods and Figures, e.g. bootstrap (the number of replicates), the type of input, and add bootstrap values to the branches, to support your separation into clades.

2, Lines 112-116: Is the LaTRE1 gene highly expressed in the roots compared to other tissues (leaves, stems, flowers) in white lupin?

3, Lines 30-32, 154 and 281: As authors write "inhibition of LaTRE1 activity by validamycin A, a potent trehalase inhibitor, resulted in the accumulation of trehalose" and "LaTRE1 is negatively associated with the formation of CRs", but the evidence is insufficient and needs to improve. The authors investigated the phenotype of LaTRE1-overexpression lines, but did not perform RNAi or knockout of this gene in the roots. Although the authors used the trehalase inhibitor ValA, this may not completely demonstrate the phenotype of LaTRE1 suppression. Because there was not direct evidence to suggest that the LaTRE1 protein has a true role of degrading trehalose into glucose, and the changes in enzyme activity after ValA treatment have not been determined.

4, Lines 163-165 and Figure 4D: Did the authors also investigate the number of CRs, fresh weight of root/shoot, and the ratio of root-to-shoot fresh biomass in the LaTRE1-overexpression composite plants? The relevant data should be provided in the Supplemental Figure.

5, Line 194 and Figure 7D: As the authors write "LaLBD16 is able to restore the density of lateral rootlet repressed by LaTRE1", please explain why type III transgenic roots with only LaLBD16 overexpression showed a slight increase in lateral rootlet density compared to the 35S::GFP controls (not significant). Considering the instability of instantaneous hairy root transformation, four independent transgenic roots were not sufficient to elucidate this phenotype. Have two independent experiments been conducted (at different times) and shown similar results? Please provide the gross morphologies of the composite plants with four types transgenic roots under P-deficiency in Figure 7 or Supplemental Figure.

6, Lines 443-444: "Differentially expressed genes (DEGs) were identified using DEseqR package with the thresholds of |log2FC| {greater than or equal to} 1, $P < 0.05$ and $FDR < 0.2$". FDR is usually set to less than 0.05.

Responses to editor:

Question#1: The authors investigated the phenotype of *LaTRE1*-overexpression lines, but did not perform RNAi or knockout of this gene in the roots. Although the authors used the trehalase inhibitor ValA, this may not completely demonstrate the phenotype of *LaTRE1* suppression. Additional genetic test is necessary.

Answer#1: Thanks for your suggestion. As shown in Fig. 8, TRV-VIGS assay was performed to suppress the expression of *LaTRE1* in white lupin roots, and we found that silencing of *LaTRE1* promoted the formation of CRs, which was similar to the phenotypes observed in ValA-treated plants under P-deficiency. These results suggested that *LaTRE1* was negatively associated with the formation of CRs in white lupin under P-deficiency.

Q#2: Is the *LaTRE1* gene highly expressed in the roots compared to other tissues (leaves, stems, flowers) in white lupin? This should be clarified.

A#2: qRT-PCR analyses revealed that *LaTRE1* was highly expressed in stems and roots, and was lowly expressed in leaves (Fig. 1F). However, given that *LaTRE1* responses to P-deficiency in roots and is differentially expressed during the development of CRs (Fig. 1F), which is the most important organ for efficient remobilization and acquisition of soil-P, we therefore mainly focused on the function of *LaTRE1* in roots in this study.

Q#3: Considering the instability of instantaneous hairy root transformation, four independent transgenic roots were not sufficient to elucidate this phenotype of *LaLBD16* overexpression. Have two independent experiments been conducted (at different times) and shown similar results? Please repeat and confirm these results.

A#3: Thanks for your suggestion. To confirm the role of *LaLBD16* in *LaTRE1*-involved CR formation, two independent TRV-VIGS assays targeting different regions of the coding sequence of *LaLBD16* were performed to silence its expression in white lupin roots. We found that the *LaLBD16*-silenced plants of both

independent lines displayed the reduction of CR formation (Fig. 9), which was opposite to the phenotypes observed in *LaTRE1*-silenced plants (Fig. 8). In combination with *LaLBD16* expression patterns in *LaTRE1*-overexpression and -suppression lines (Fig. 6B; Supplemental Fig. S6), it has been suggested that *LaTRE1* negatively regulates the formation of CRs, at least partially, by the down-regulation of *LaLBD16*.

Responses to Reviewer #1:

Q#1: P deficiency induces the expression of *LaTRE1* and the formation CRs, whereas *LaTRE1* would repress the formation of CRs. How to explain this discrepancy?

A#1: First, overexpression of *LaTRE1* reduced the density of lateral rootlet of hairy roots (Fig. 4F) and silencing of *LaTRE1* induced the number of CRs (Fig. 8), suggesting that *LaTRE1* is indeed negatively associated with the formation of CRs. Second, detailedly analyzing the expression of *LaTRE1* in different types of roots, we found that *LaTRE1* is induced by P-deficiency in normal roots, however, during the development of CRs, *LaTRE1* was lower expressed in the pre-emergent zone in which the lateral rootlet primordia of CRs initiation occurs, and highest expressed at the maturation stage of CRs than that in +PNR (Fig. 1F). These results suggested that lower expression of *LaTRE1* is required for the initiation of CRs.

Q#2: The authors used ValA to inhibit the activity of trehalase to investigate the role of *LaTRE1*. It needs to show the data that the activity of trehalase is really and specially repressed.

A#2: Thanks for your suggestion. The activities of trehalase were determined in ValA-treated and -untreated roots of white lupin under P-sufficient and -deficient conditions. As shown in Supplemental Fig. S4, ValA treatment significantly inhibited the activities of trehalase in white lupin roots under P-sufficient and -deficient conditions, which is consistent with the previous reports in *Arabidopsis*, *M. truncatula* and soybean (Müller et al., 1995; Muller et al., 2001; López et al., 2009).

Q#3: The data of CRs in the *LaTRE1* over-expression transgenic plants is not shown in this manuscript. Can the authors give some explanations?

A#3: The *A. rhizogenes*-based transformation resulted in the development of "hairy-root" phenotype characterized by a high degree of lateral root branching (Chilton et al., 1982; Collier et al., 2005), which makes us difficult to visually

determine the typical CR phenotypes as shown in Fig. 1E. Thus, only the lateral rootlet length and density of transgenic roots were analyzed.

Q#4: The authors showed that *LaLBD16* might be an important downstream gene in mediating *LaTRE1*-modulated root development. It would be necessary to examine the expression of *LaLBD16* under ValA treatment, which would further support the regulatory role of *LaTRE1*.

A#4: To better understand the role of *LaLBD16* in *LaTRE1*-modulated CR formation, the TRV-VIGS assays were performed to silence *LaTRE1* and *LaLBD16* expression in white lupin roots respectively. We found that silencing of *LaTRE1* led to the induction of *LaLBD16* expression and CR formation (Fig. 8), by contrast, silencing of *LaLBD16* resulted in the reduction of CR formation (Fig. 9), supporting the vital role of *LaLBD16* in mediating *LaTRE1*-modulated CR formation.

Responses to Reviewer #2:

Q#1: Lines 105-109, 367-368 and Figures 1B, S1: The phylogenetic tree is not sufficient to clearly separate different clades, because of missing phylogenetic information. Please provide detailed information on the settings in the Materials & Methods and Figures, e.g. bootstrap (the number of replicates), the type of input, and add bootstrap values to the branches, to support your separation into clades.

A#1: Thanks for your suggestions, the detailed phylogenetic information had been provided in Fig. 1B, Supplemental Fig. S1 and Materials and Methods.

Q#2: Lines 112-116: Is the *LaTRE1* gene highly expressed in the roots compared to other tissues (leaves, stems, flowers) in white lupin?

A#2: As shown in Fig. 1F, *LaTRE1* was highly expressed in stems, followed by roots, and was lowly expressed in leaves. Given that *LaTRE1* responses to P-deficiency in roots and is differentially expressed during the CR development, the most important organ for efficient acquisition of soil-P, we therefore mainly focused on the function of *LaTRE1* in roots in this study.

Q#3: Lines 30-32, 154 and 281: As authors write "inhibition of LaTRE1 activity by validamycin A, a potent trehalase inhibitor, resulted in the accumulation of trehalose" and "*LaTRE1* is negatively associated with the formation of CRs", but the evidence is insufficient and needs to improve. The authors investigated the phenotype of *LaTRE1*-overexpression lines, but did not perform RNAi or knockout of this gene in the roots. Although the authors used the trehalase inhibitor ValA, this may not completely demonstrate the phenotype of *LaTRE1* suppression. Because there was no direct evidence to suggest that the LaTRE1 protein has a true role of degrading trehalose into glucose, and the changes in enzyme activity after ValA treatment have not been determined.

A#3: First, protein sequence alignment and structure prediction revealed that LaTRE1 showed a high homology with AtTRE1 (Fig. 1C; Supplemental Fig. S2), which can

functionally complement the phenotype of yeast acid trehalase loss-of-function mutant (Frison et al., 2007). Second, ValA treatment inhibited the activities of trehalase and promoted the accumulation of trehalose in white lupin roots (Supplemental Fig. S4; Fig. 3A). Third, silencing of *LaTRE1* or ValA treatment to inhibit trehalase resulted in the induction of CR formation (Figs. 2 and 8), while overexpression of *LaTRE1* led to the reduction of trehalose and lateral rootlet density of hairy roots (Figs. 4F and 5C). Together, all results suggested that *LaTRE1* is involved in trehalose metabolism and negatively associated with the formation of CRs.

Q#4: Lines 163-165 and Figure 4D: Did the authors also investigate the number of CRs, fresh weight of root/shoot, and the ratio of root-to-shoot fresh biomass in the *LaTRE1*-overexpression composite plants? The relevant data should be provided in the Supplemental Figure.

A#4: As mentioned above, *A. rhizogenes*-based transformation led to the development of "hairy-root" phenotype characterized with high degrees of lateral branching (Chilton et al., 1982; Collier et al., 2005), which makes us difficult to determine the typical CR phenotypes as shown in Fig. 1E. Therefore, only the lateral rootlet length and density of transgenic hairy roots were analyzed.

However, we investigated the number of CRs, fresh weight of root/shoot, and the ratio of root-to-shoot fresh biomass in the *LaTRE1*-silenced plants (Fig. 8), which were similar to the phenotypes of ValA-treatment plants (Fig. 2).

Q#5: Line 194 and Figure 7D: As the authors write "*LaLBD16* is able to restore the density of lateral rootlet repressed by *LaTRE1*", please explain why type III transgenic roots with only *LaLBD16* overexpression showed a slight increase in lateral rootlet density compared to the *35S::GFP* controls (not significant). Considering the instability of instantaneous hairy root transformation, four independent transgenic roots were not sufficient to elucidate this phenotype. Have two independent experiments been conducted (at different times) and shown similar results? Please

provide the gross morphologies of the composite plants with four types transgenic roots under P-deficiency in Figure 7 or Supplemental Figure.

A#5: Thanks for your suggestion. To overcome the shortcoming of *A. rhizogenes*-based hairy root transformation, an alternative TRV-VIGS approach was carried out to better investigate the role of *LaLBD16* in *LaTRE1*-involved CR formation. We found that silencing of *LaTRE1* led to the induction of *LaLBD16* expression and CR formation (Fig. 8; Supplemental Fig. S6), by contrast, silencing of *LaLBD16* induced the number of CRs (Fig. 9). Two independent TRV-VIGS assays targeting different regions of the coding sequences of *LaTRE1* and *LaLBD16* genes were conducted and similar results were obtained.

Q#6: Lines 443-444: "Differentially expressed genes (DEGs) were identified using DEseqR package with the thresholds of $|\log_2 FC| \geq 1$, $P < 0.05$ and FDR < 0.2". FDR is usually set to less than 0.05.

A#6: We agreed with the reviewer's opinion that FDR is usually set to < 0.05. However, differential expression analysis between *35S::LaTRE1* and *35S::GFP* showed 98 genes changing at FDR < 0.05, 146 genes at FDR < 0.1, and 231 genes at FDR < 0.2. To yield a sufficient number of DEGs for further analyses, we used a more lenient cutoff, FDR < 0.2. To reduce the risk of false positives, qRT-PCR validation of key genes was conducted.

Reference

Chilton M-D, Tepfer DA, Petit A, David C, Casse-Delbart F, Tempé J (1982) Agrobacterium rhizogenes inserts T-DNA into the genomes of the host plant root cells. Nature **295:** 432-434

Collier R, Fuchs B, Walter N, Kevin Lutke W, Taylor CG (2005) Ex vitro composite plants: an inexpensive, rapid method for root biology. The Plant Journal **43:** 449-457

Frison M, Parrou JL, Guillaumot D, Masquelier D, François J, Chaumont F, Batoko H (2007) The Arabidopsis thaliana trehalase is a plasma membrane-bound enzyme with extracellular activity. FEBS Letters **581:** 4010-4016

López M, Tejera NA, Lluch C (2009) Validamycin A improves the response of Medicago truncatula plants to salt stress by inducing trehalose accumulation in the root nodules. Journal of Plant Physiology **166:** 1218-1222

Muller J, Aeschbacher RA, Wingler A, Boller T, Wiemken A (2001) Trehalose and trehalase in Arabidopsis. Plant physiology **125:** 1086-1093

Müller J, Boller T, Wiemken A (1995) Effects of validamycin A, a potent trehalase inhibitor, and phytohormones on trehalose metabolism in roots and root nodules of soybean and cowpea. Planta **197:** 362-368

11-Apr-2024

Prof. Dr. Weifeng Xu
Fujian Agriculture and Forestry University
Jinshan Fuzhou, orcid||||||
China

RE: Trehalase gene LaTRE1 is important for the formation and function of cluster roots in white lupin under P-deficiency

Dear Dr. Xu:

We have received reviews of your manuscript, which you may view at the end of this letter. On the basis of the advice received and my own evaluation, I believe that, pending revision, this article may ultimately be acceptable for publication in Plant Physiology.

There are some concerns to be addressed:
1. Transgenic hairy roots are not the best way to study gene function because of temporary expression of target genes. Did the authors ever try the stable transformation of white lupin? That should be explained in the text.
2. From the Fig1B, we can see TRE1 homologous genes in G.max, M.truncatula and P. valgaris, all are legume plants. What function would these homologues have in these plants? These should be discussed.
3. A recent work by Yang et al (2023) doi.org/10.1093/plphys/kiad570 found that modifying GmRR1 expression enhanced auxin and cytokinin levels in roots, promoting increases in root hairs, root length, and root surface area in response to low P stress. Is this comparable to the finding of this manuscript? This could be discussed.

When you are ready to submit your revision, click the below link and find the red arrow under your Author Tasks heading for this revision submission.

If you want to visit this version of the manuscript, you may click the link below and then click on the folder that reads "Post Decision Manuscripts".

Link Not Available

When uploading the revised version of this article, please be sure to include as complete an answer as possible to the referees' criticisms during the first round of reviews in the "response to reviewers" section.

Plant Physiology considers a revision for up to 60 days after a manuscript has been provisionally accepted; limited extension of this timeframe is at the discretion of the Monitoring Editor. A reminder notice will be sent to you 2 weeks before the deadline, at which time you may choose to contact the editorial office to request an extension; if so, please provide a justification for your request. After the expiration of this deadline, the article would need to be submitted as a resubmission and would be handled as such.

Thank you very much for giving us an opportunity to review this work. I look forward to receiving the next version.

Sincerely,

Editor, Plant Physiology

Want to add this revision deadline to your calendar? Click below!

-- Reviewer comments:
Reviewer #1 (Comments for the Author):

The newly submitted version has been substantially improved, and all of my concerns also are answered properly. I have no

further questions, and totally support for its publication in the journal.

Reviewer #2 (Comments for the Author):

This manuscript has been improved. Still, there are some concerns to be addressed.

1. Transgenic hairy roots are not the best way to study gene function because of temporary expression of target genes. Did the authors ever try the stable transformation of white lupin? That should be explained in the text.
2. From the Fig1B, we can see TRE1 homologous genes in G.max, M.truncatula and P. valgaris, all are legume plants. What function would these homologues have in these plants? These should be discussed.
3. A recent work by Yang et al (2023) doi.org/10.1093/plphys/kiad570 found that modifying GmRR1 expression enhanced auxin and cytokinin levels in roots, promoting increases in root hairs, root length, and root surface area in response to low P stress. Is this comparable to the finding of this manuscript? This could be discussed.

First round of reviews:

Responses to editor:

Question#1: The authors investigated the phenotype of *LaTRE1*-overexpression lines, but did not perform RNAi or knockout of this gene in the roots. Although the authors used the trehalase inhibitor ValA, this may not completely demonstrate the phenotype of *LaTRE1* suppression. Additional genetic test is necessary.

Answer#1: Thanks for your suggestion. As shown in Fig. 8, TRV-VIGS assay was performed to suppress the expression of *LaTRE1* in white lupin roots, and we found that silencing of *LaTRE1* promoted the formation of CRs, which was similar to the phenotypes observed in ValA-treated plants under P-deficiency. These results suggested that *LaTRE1* was negatively associated with the formation of CRs in white lupin under P-deficiency.

Q#2: Is the *LaTRE1* gene highly expressed in the roots compared to other tissues (leaves, stems, flowers) in white lupin? This should be clarified.

A#2: qRT-PCR analyses revealed that *LaTRE1* was highly expressed in stems and roots, and was lowly expressed in leaves (Fig. 1F). However, given that *LaTRE1* responses to P-deficiency in roots and is differentially expressed during the development of CRs (Fig. 1F), which is the most important organ for efficient remobilization and acquisition of soil-P, we therefore mainly focused on the function of *LaTRE1* in roots in this study.

Q#3: Considering the instability of instantaneous hairy root transformation, four independent transgenic roots were not sufficient to elucidate this phenotype of *LaLBD16* overexpression. Have two independent experiments been conducted (at different times) and shown similar results? Please repeat and confirm these results.

A#3: Thanks for your suggestion. To confirm the role of *LaLBD16* in *LaTRE1*-involved CR formation, two independent TRV-VIGS assays targeting

different regions of the coding sequence of *LaLBD16* were performed to silence its expression in white lupin roots. We found that the *LaLBD16*-silenced plants of both independent lines displayed the reduction of CR formation (Fig. 9), which was opposite to the phenotypes observed in *LaTRE1*-silenced plants (Fig. 8). In combination with *LaLBD16* expression patterns in *LaTRE1*-overexpression and -suppression lines (Fig. 6B; Supplemental Fig. S6), it has been suggested that *LaTRE1* negatively regulates the formation of CRs, at least partially, by the down-regulation of *LaLBD16*.

Responses to Reviewer #1:

Q#1: P deficiency induces the expression of *LaTRE1* and the formation CRs, whereas *LaTRE1* would repress the formation of CRs. How to explain this discrepancy?

A#1: First, overexpression of *LaTRE1* reduced the density of lateral rootlet of hairy roots (Fig. 4F) and silencing of *LaTRE1* induced the number of CRs (Fig. 8), suggesting that *LaTRE1* is indeed negatively associated with the formation of CRs. Second, detailedly analyzing the expression of *LaTRE1* in different types of roots, we found that *LaTRE1* is induced by P-deficiency in normal roots, however, during the development of CRs, *LaTRE1* was lower expressed in the pre-emergent zone in which the lateral rootlet primordia of CRs initiation occurs, and highest expressed at the maturation stage of CRs than that in +PNR (Fig. 1F). These results suggested that lower expression of *LaTRE1* is required for the initiation of CRs.

Q#2: The authors used ValA to inhibit the activity of trehalase to investigate the role of *LaTRE1*. It needs to show the data that the activity of trehalase is really and specially repressed.

A#2: Thanks for your suggestion. The activities of trehalase were determined in ValA-treated and -untreated roots of white lupin under P-sufficient and -deficient conditions. As shown in Supplemental Fig. S4, ValA treatment significantly inhibited the activities of trehalase in white lupin roots under P-sufficient and -deficient

conditions, which is consistent with the previous reports in *Arabidopsis*, *M. truncatula* and soybean (Müller et al., 1995; Muller et al., 2001; López et al., 2009).

Q#3: The data of CRs in the *LaTRE1* over-expression transgenic plants is not shown in this manuscript. Can the authors give some explanations?

A#3: The *A. rhizogenes*-based transformation resulted in the development of "hairy-root" phenotype characterized by a high degree of lateral root branching (Chilton et al., 1982; Collier et al., 2005), which makes us difficult to visually determine the typical CR phenotypes as shown in Fig. 1E. Thus, only the lateral rootlet length and density of transgenic roots were analyzed.

Q#4: The authors showed that *LaLBD16* might be an important downstream gene in mediating *LaTRE1*-modulated root development. It would be necessary to examine the expression of *LaLBD16* under ValA treatment, which would further support the regulatory role of *LaTRE1*.

A#4: To better understand the role of *LaLBD16* in *LaTRE1*-modulated CR formation, the TRV-VIGS assays were performed to silence *LaTRE1* and *LaLBD16* expression in white lupin roots respectively. We found that silencing of *LaTRE1* led to the induction of *LaLBD16* expression and CR formation (Fig. 8), by contrast, silencing of *LaLBD16* resulted in the reduction of CR formation (Fig. 9), supporting the vital role of *LaLBD16* in mediating *LaTRE1*-modulated CR formation.

Responses to Reviewer #2:

Q#1: Lines 105-109, 367-368 and Figures 1B, S1: The phylogenetic tree is not sufficient to clearly separate different clades, because of missing phylogenetic information. Please provide detailed information on the settings in the Materials & Methods and Figures, e.g. bootstrap (the number of replicates), the type of input, and add bootstrap values to the branches, to support your separation into clades.

A#1: Thanks for your suggestions, the detailed phylogenetic information had been provided in Fig. 1B, Supplemental Fig. S1 and Materials and Methods.

Q#2: Lines 112-116: Is the *LaTRE1* gene highly expressed in the roots compared to other tissues (leaves, stems, flowers) in white lupin?

A#2: As shown in Fig. 1F, *LaTRE1* was highly expressed in stems, followed by roots, and was lowly expressed in leaves. Given that *LaTRE1* responses to P-deficiency in roots and is differentially expressed during the CR development, the most important organ for efficient acquisition of soil-P, we therefore mainly focused on the function of *LaTRE1* in roots in this study.

Q#3: Lines 30-32, 154 and 281: As authors write "inhibition of LaTRE1 activity by validamycin A, a potent trehalase inhibitor, resulted in the accumulation of trehalose" and "*LaTRE1* is negatively associated with the formation of CRs", but the evidence is insufficient and needs to improve. The authors investigated the phenotype of *LaTRE1*-overexpression lines, but did not perform RNAi or knockout of this gene in the roots. Although the authors used the trehalase inhibitor ValA, this may not completely demonstrate the phenotype of *LaTRE1* suppression. Because there was no direct evidence to suggest that the LaTRE1 protein has a true role of degrading trehalose into glucose, and the changes in enzyme activity after ValA treatment have not been determined.

A#3: First, protein sequence alignment and structure prediction revealed that LaTRE1 showed a high homology with AtTRE1 (Fig. 1C; Supplemental Fig. S2), which can functionally complement the phenotype of yeast acid trehalase loss-of-function mutant (Frison et al., 2007). Second, ValA treatment inhibited the activities of trehalase and promoted the accumulation of trehalose in white lupin roots (Supplemental Fig. S4; Fig. 3A). Third, silencing of *LaTRE1* or ValA treatment to inhibit trehalase resulted in the induction of CR formation (Figs. 2 and 8), while overexpression of *LaTRE1* led to the reduction of trehalose and lateral rootlet density of hairy roots (Figs. 4F and 5C). Together, all results suggested that *LaTRE1* is involved in trehalose metabolism and negatively associated with the formation of CRs.

Q#4: Lines 163-165 and Figure 4D: Did the authors also investigate the number of CRs, fresh weight of root/shoot, and the ratio of root-to-shoot fresh biomass in the *LaTRE1*-overexpression composite plants? The relevant data should be provided in the Supplemental Figure.

A#4: As mentioned above, *A. rhizogenes*-based transformation led to the development of "hairy-root" phenotype characterized with high degrees of lateral branching (Chilton et al., 1982; Collier et al., 2005), which makes us difficult to determine the typical CR phenotypes as shown in Fig. 1E. Therefore, only the lateral rootlet length and density of transgenic hairy roots were analyzed.

However, we investigated the number of CRs, fresh weight of root/shoot, and the ratio of root-to-shoot fresh biomass in the *LaTRE1*-silenced plants (Fig. 8), which were similar to the phenotypes of ValA-treatment plants (Fig. 2).

Q#5: Line 194 and Figure 7D: As the authors write "*LaLBD16* is able to restore the density of lateral rootlet repressed by *LaTRE1*", please explain why type III transgenic roots with only *LaLBD16* overexpression showed a slight increase in lateral rootlet density compared to the *35S::GFP* controls (not significant). Considering the instability of instantaneous hairy root transformation, four independent transgenic roots were not sufficient to elucidate this phenotype. Have two independent experiments been conducted (at different times) and shown similar results? Please provide the gross morphologies of the composite plants with four types transgenic roots under P-deficiency in Figure 7 or Supplemental Figure.

A#5: Thanks for your suggestion. To overcome the shortcoming of *A. rhizogenes*-based hairy root transformation, an alternative TRV-VIGS approach was carried out to better investigate the role of *LaLBD16* in *LaTRE1*-involved CR formation. We found that silencing of *LaTRE1* led to the induction of *LaLBD16* expression and CR formation (Fig. 8; Supplemental Fig. S6), by contrast, silencing of *LaLBD16* induced the number of CRs (Fig. 9). Two independent TRV-VIGS assays

targeting different regions of the coding sequences of *LaTRE1* and *LaLBD16* genes were conducted and similar results were obtained.

Q#6: Lines 443-444: "Differentially expressed genes (DEGs) were identified using DEseqR package with the thresholds of $|\log_2 FC| \geq 1$, $P < 0.05$ and FDR < 0.2". FDR is usually set to less than 0.05.

A#6: We agreed with the reviewer's opinion that FDR is usually set to < 0.05. However, differential expression analysis between *35S::LaTRE1* and *35S::GFP* showed 98 genes changing at FDR < 0.05, 146 genes at FDR < 0.1, and 231 genes at FDR < 0.2. To yield a sufficient number of DEGs for further analyses, we used a more lenient cutoff, FDR < 0.2. To reduce the risk of false positives, qRT-PCR validation of key genes was conducted.

Reference

Chilton M-D, Tepfer DA, Petit A, David C, Casse-Delbart F, Tempé J (1982) Agrobacterium rhizogenes inserts T-DNA into the genomes of the host plant root cells. Nature **295:** 432-434

Collier R, Fuchs B, Walter N, Kevin Lutke W, Taylor CG (2005) Ex vitro composite plants: an inexpensive, rapid method for root biology. The Plant Journal **43:** 449-457

Frison M, Parrou JL, Guillaumot D, Masquelier D, François J, Chaumont F, Batoko H (2007) The Arabidopsis thaliana trehalase is a plasma membrane-bound enzyme with extracellular activity. FEBS Letters **581:** 4010-4016

López M, Tejera NA, Lluch C (2009) Validamycin A improves the response of Medicago truncatula plants to salt stress by inducing trehalose accumulation in the root nodules. Journal of Plant Physiology **166:** 1218-1222

Muller J, Aeschbacher RA, Wingler A, Boller T, Wiemken A (2001) Trehalose and trehalase in Arabidopsis. Plant physiology **125:** 1086-1093

Müller J, Boller T, Wiemken A (1995) Effects of validamycin A, a potent trehalase inhibitor, and phytohormones on trehalose metabolism in roots and root nodules of soybean and cowpea. Planta **197:** 362-368

Second round of reviews:

Responses to editor:

Question#1: Transgenic hairy roots are not the best way to study gene function because of temporary expression of target genes. Did the authors ever try the stable transformation of white lupin? That should be explained in the text.

Answer#1: Currently, no stable transformation protocol was successfully reported in white lupin. We didn't obtain the stable *LaTRE1*-overexpressing or -silencing plants of white lupin either, because of the failure of differentiation from the transgenic roots to whole plants. We have explained it in Discussion section.

Q#2: From the Fig1B, we can see *TRE1* homologous genes in *G. max*, *M. truncatula* and *P. valgaris*, all are legume plants. What function would these homologues have in these plants? These should be discussed.

A#2: Thanks for your suggestion. Several studies shown that *TRE1* genes in *G. max*, *M. truncatula* and *P. valgaris* were involved in the regulation of trehalose contents, nodule development and salt stress response (Aeschbacher et al., 1999; López et al., 2008; López et al., 2009; Barraza et al., 2013). We have discussed it in Discussion section.

Q#3: A recent work by Yang et al (2023) doi.org/10.1093/plphys/kiad570 found that modifying *GmRR1* expression enhanced auxin and cytokinin levels in roots, promoting increases in root hairs, root length, and root surface area in response to low P stress. Is this comparable to the finding of this manuscript? This could be discussed.

A#3: Yes. In consistent with the findings in (Yang et al., 2024), we found that *LaTRE1* negatively regulates the formation of CRs probably via a mechanism involving auxin. We have discussed it in Discussion section.

Responses to Reviewer #1:

Q#1: The newly submitted version has been substantially improved, and all of my concerns also are answered properly. I have no further questions, and totally support for its publication in the journal.

A#1: Many thanks for your comments.

Responses to Reviewer #2:

Question#1: Transgenic hairy roots are not the best way to study gene function because of temporary expression of target genes. Did the authors ever try the stable transformation of white lupin? That should be explained in the text.

Answer#1: Currently, no stable transformation protocol was successfully reported in white lupin. We didn't obtain the stable *LaTRE1*-overexpressing or -silencing plants of white lupin either, because of the failure of differentiation from the transgenic roots to whole plants. We have explained it in Discussion section.

Q#2: From the Fig1B, we can see TRE1 homologous genes in *G.max*, *M.truncatula* and *P. valgaris*, all are legume plants. What function would these homologues have in these plants? These should be discussed.

A#2: Thanks for your suggestion. Several studies shown that *TRE1* genes in *G. max*, *M. truncatula* and *P. valgaris* were involved in the regulation of trehalose contents, nodule development and salt stress response (Aeschbacher et al., 1999; López et al., 2008; López et al., 2009; Barraza et al., 2013). We have discussed it in Discussion section.

Q#3: A recent work by Yang et al (2023) doi.org/10.1093/plphys/kiad570 found that modifying *GmRR1* expression enhanced auxin and cytokinin levels in roots, promoting increases in root hairs, root length, and root surface area in response to low P stress. Is this comparable to the finding of this manuscript? This could be discussed.

A#3: Yes. In consistent with the findings in (Yang et al., 2024), we found that *LaTRE1* negatively regulates the formation of CRs probably via a mechanism involving auxin. We have discussed it in Discussion section.

Reference:

Aeschbacher RA, Müller J, Boller T, Wiemken A (1999) Purification of the Trehalase GMTRE1 from Soybean Nodules and Cloning of Its cDNA. GMTRE1 Is Expressed at a Low Level in Multiple Tissues1. Plant Physiology **119:** 489-496

Barraza A, Estrada-Navarrete G, Rodriguez-Alegria ME, Lopez-Munguia A, Merino E, Quinto C, Sanchez F (2013) Down-regulation of PvTRE1 enhances nodule biomass and bacteroid number in the common bean. New Phytologist **197:** 194-206

López M, Tejera NA, Iribarne C, Lluch C, Herrera-Cervera JA (2008) Trehalose and trehalase in root nodules of Medicago truncatula and Phaseolus vulgaris in response to salt stress. Physiologia Plantarum **134:** 575-582

López M, Tejera NA, Lluch C (2009) Validamycin A improves the response of Medicago truncatula plants to salt stress by inducing trehalose accumulation in the root nodules. Journal of Plant Physiology **166:** 1218-1222

Yang Y, Wang L, Zhang D, Che Z, Wang Q, Cui R, Zhao W, Huang F, Zhang H, Cheng H, Yu D (2024) Soybean type-B response regulator GmRR1 mediates phosphorus uptake and yield by modifying root architecture. Plant Physiol **194:** 1527-1544

29-Apr-2024
Prof. Dr. Weifeng Xu
福建农林大学
Jinshan Fuzhou, orcid||||||
China

MSID: PP2024-RA-00452DR1
MS TITLE: Trehalase gene LaTRE1 is important for the formation and function of cluster roots in white lupin under P-deficiency

Dear Dr. Weifeng Xu:

I am pleased to inform you that your revised manuscript "Trehalase gene LaTRE1 is important for the formation and function of cluster roots in white lupin under P-deficiency" has been accepted for publication in Plant Physiology. Your manuscript will be checked for consistency with journal formatting and image standards by one of our Science Editors, who will contact you shortly. They may have a number of queries and suggestions for you. Please keep in mind that they work to increase the online visibility of your article and its attraction for readers from the widest of backgrounds. You will have the opportunity to review any revisions, and I hope you will work constructively with them to get the most out of your research. You will also have the opportunity to review all changes when you receive the author proofs for your article.

Once validated for publication, your article will be sent to a Science Editor for a final review of your manuscript and source files. After your final files are uploaded, your article will appear in the next available issue and the online Preview version of your article will be posted after your signed Copyright and Checklist forms and final source files are received.

Please be sure to include the following in your manuscript file:
Harvard style citations
List of all author contributions and funding information at the beginning of manuscript after the titles and author list.
List all supplemental materials associated with the article, with titles, at the end of the ms, before the references and acknowledgments section.
Complete Figure Legends for all figures appearing in the article listed before the Literature Cited.

Thank you and congratulations on your Plant Physiology paper!

Sincerely,
ditor, Plant Physiology

=====
FIRST AUTHOR PROFILES
=====

We recognize the hard work that goes into being First Author of a Plant Physiology paper by publishing a "First Author Feature" for each First Author alongside your paper.

If the first author(s) would like to submit information for a feature on Facebook and Twitter, fill out the form by clicking on the link below:

Plant Physiology First Author Feature Form: https://forms.office.com/r/ZBCGZfhm8F
If you cannot access the form through the link above, please contact Rachel at rbelsky@aspb.org.

To upload an image of yourself to accompany your profile, please put your photo in the correct journal folder at the link below. You must label your image: Author First Name Last Name Article Title.

First Author Profile Image Dropbox: https://www.dropbox.com/scl/fo/xz029p7qjr2jml2rp2kmq/h?dl=0&rlkey=6fkxcaqmxx35ap152b2osy1fz

=====
IMPORTANT REMINDER: PEER REVIEW REPORTS

=====

If you opted to publish a peer review report along with your article during the original submission process, it will be prepared by the editorial staff and publicly posted with your manuscript, inside the zip file that contains any other supplemental material. As a reminder, the peer review report is a public record of all comments from editors and reviewers, as well as your prior responses, as you received them in the decision letters for each draft of your manuscript. If you agreed to publish this report and have changed your mind, or are not sure if you selected this option, please contact the editorial office as soon as possible before signing the license agreement from our publisher.

-- Reviewer comments:
